# The role of Lon-mediated proteolysis in the dynamics of mitochondrial nucleic acid-protein complexes

**DOI:** 10.1038/s41598-017-00632-8

**Published:** 2017-04-04

**Authors:** Nina Kunová, Gabriela Ondrovičová, Jacob A. Bauer, Jana Bellová, Ľuboš Ambro, Lucia Martináková, Veronika Kotrasová, Eva Kutejová, Vladimír Pevala

**Affiliations:** 1 Department of Biochemistry and Structural Biology, Institute of Molecular Biology, Slovak Academy of Sciences, Dúbravská cesta 21, 845 51 Bratislava, Slovakia; 2 Institute of Microbiology, Academy of Sciences of the Czech Republic, BIOCEV, Průmyslová 595, 252 42 Vestec, Czech Republic; 3 Pavol Jozef Šafárik University in Košice, Faculty of Medicine, Trieda SNP 1, 040 11 Košice, Slovakia

## Abstract

Mitochondrial nucleoids consist of several different groups of proteins, many of which are involved in essential cellular processes such as the replication, repair and transcription of the mitochondrial genome. The eukaryotic, ATP-dependent protease Lon is found within the central nucleoid region, though little is presently known about its role there. Aside from its association with mitochondrial nucleoids, human Lon also specifically interacts with RNA. Recently, Lon was shown to regulate TFAM, the most abundant mtDNA structural factor in human mitochondria. To determine whether Lon also regulates other mitochondrial nucleoid- or ribosome-associated proteins, we examined the *in vitro* digestion profiles of the *Saccharomyces cerevisiae* TFAM functional homologue Abf2, the yeast mtDNA maintenance protein Mgm101, and two human mitochondrial proteins, Twinkle helicase and the large ribosomal subunit protein MrpL32. Degradation of Mgm101 was also verified *in vivo* in yeast mitochondria. These experiments revealed that all four proteins are actively degraded by Lon, but that three of them are protected from it when bound to a nucleic acid; the Twinkle helicase is not. Such a regulatory mechanism might facilitate dynamic changes to the mitochondrial nucleoid, which are crucial for conducting mitochondrial functions and maintaining mitochondrial homeostasis.

## Introduction

Mitochondria are some of the most important organelles of eukaryotic cells. Their proper function depends on both nuclear and mitochondrial DNA, whose coordinated regulation of transcription and translation is crucial for cellular survival^1^. Nuclear DNA is commonly organized into a nucleoprotein structure called chromatin, while mitochondrial DNA (mtDNA) exists as nucleoids, somewhat less well-organized protein–DNA complexes that more closely resemble the bacterial nucleoid rather than nuclear chromatin. Architecturally, these nucleoids consist of a relatively compact core, containing a number of packaging proteins, and a looser periphery, which is bound by a number of important regulatory and signalling proteins^1^.

The principal protein component of the nucleoids found in mammalian mitochondria is the DNA packaging protein TFAM (Transcription Factor A, Mitochondrial), a high mobility group (HMG) protein^2, 3^. It has been shown that the level of TFAM is linked to the mtDNA copy number^4–6^ and that the protein has vital roles in mtDNA replication and gene expression^7^. Fluctuations in TFAM levels have also been linked with changes in nucleoid morphology^8^. A given mitochondrion often contains more than one nucleoid, and these nucleoids have been observed in two forms: a highly compacted form, which is presumably used for DNA storage, and a more elongated form, where replication and translation are probably occurring^8^. TFAM is released from DNA upon phosphorylation by protein kinase A, and TFAM not bound to DNA, whether phosphorylated or not, is a substrate for the ATP-dependent mitochondrial Lon protease^9^. By controlling the levels of unassociated TFAM, Lon is one of the agents responsible for controlling mitochondrial nucleoid dynamics^4, 5^. There are other proteins which influence nucleoid dynamics, however, most obviously those responsible for mtDNA replication, repair, recombination, and transcription, along with some needed for mtDNA packaging, division, and sorting^10^.

The mitochondrial Lon protease itself has been identified as an integral nucleoid core factor in human mitochondria^11^, it is involved in selective protein turnover (including ribosomal proteins)^12^ and the ATP-dependent degradation of misfolded or damaged mitochondrial proteins^13, 14^. It also has a chaperone-like function in the assembly of certain mitochondrial complexes, which persists even if its proteolytic activity is impaired^15^. Structurally, Lon is a dynamic protease with three functional domains (N-terminal, ATPase and proteolytic) on a single polypeptide chain^16^. To date, no complete 3D structure of mitochondrial Lon protease has been determined, although several X-ray crystal structures of separate domains are available^17^; recently a full-length cryo-electron microscopy structure of human Lon was determined, which showed the importance of the N-terminal domain for protease integrity^18^. *In vivo* studies have shown that disruption of the *LON* gene in yeast is not lethal, but does led to an inability to synthesize respiratory chain subunits, the destabilization of the mitochondrial genome, the impairment of mitochondrial gene expression, and respiratory defects^12, 19^. Thin-section electron microscopy revealed that these cells tend to accumulate electron-dense inclusion bodies in the matrix space, which likely correspond to aggregated mitochondrial proteins^12^. Mammalian cells lacking functional Lon proteases were also observed to behave in a similar manner. These cells exhibited impaired mitochondrial respiration and reduced membrane potential, resulting in diverse phenotypes depending on the organism and cell type^19, 20^.

Many substrates have been identified for this protease. In *Saccharomyces cerevisiae*, these include the mitochondrial metabolic enzymes Ilv1, Ilv2, Lsc1, Lys4, and Aco1; the α, β, and γ subunits of the F_1_F_0_ ATPase complex; both α and β subunits of the mitochondrial processing peptidase (individually, but not when combined in the MPPαβ heterodimer); and a number of mitochondrial ribosomal proteins^21–23^. The recent proteomic study of Bayot *et al*.^24^ on Lon-depleted yeast cells identified several additional potential Lon substrates, which accumulated or appeared in oxidized forms in these cells, including three components of the pyruvate dehydrogenase complex (Pdb1, Lat1, and Lpd1), six other mitochondrial metabolic enzymes (Kgd2, CoQ5, Dld1, Ald4, Aim17, Ilv5), and four respiratory chain complex subunits (Qcr2, Rip1, Cox4, and Atp7). Fewer substrates are presently known from mammalian mitochondria, but they include important proteins like aconitase, the steroidogenic acute regulatory protein (StAR), and TFAM^4, 20, 25^.

In this study, we evaluate the potential of proteins known or expected to be involved in mtDNA packaging, replication, repair, and recombination to be substrates for the mitochondrial Lon protease. Both human and yeast proteins were assessed to determine how general Lon’s influence is likely to be across even quite unrelated eukaryotic species. In particular, we studied the primary yeast mtDNA packaging protein Abf2 and compared its behaviour to that of its human counterpart TFAM. We examined the ability of Lon to degrade the human Twinkle helicase, which forms the mtDNA replication complex together with Polγ^26^. We also considered yeast Mgm101 and human MrpL32. Some forms of the yeast mtDNA-binding protein Mgm101 have been shown to carry out the Rad52 DNA recombination activities of strand annealing and D-loop formation^27, 28^, while human MrpL32, a component of the 39S large subunit of the mitochondrial ribosome, is known to be important for proper ribosome assembly and, consequently, the proper synthesis and assembly of the mtDNA-encoded components of the oxidative phosphorylation respiratory complex^29^.

For each protein, the ability of single-stranded DNA (ssDNA), double-stranded DNA (dsDNA), or RNA (in the case of MrpL32) to protect it from degradation by Lon was explored. To verify that each protein was properly folded, DNA-binding and functional assays, where appropriate, were carried out. We found that all of these proteins could serve as Lon substrates, suggesting that the mitochondrial Lon protease could be involved in the regulation of such fundamental processes as nucleoid packaging, mtDNA replication, mtDNA maintenance and recombination, and the assembly of mitochondrial ribosomes.

## Results

### Yeast Lon protease localizes to mitochondrial nucleoids

**Figure 1:**
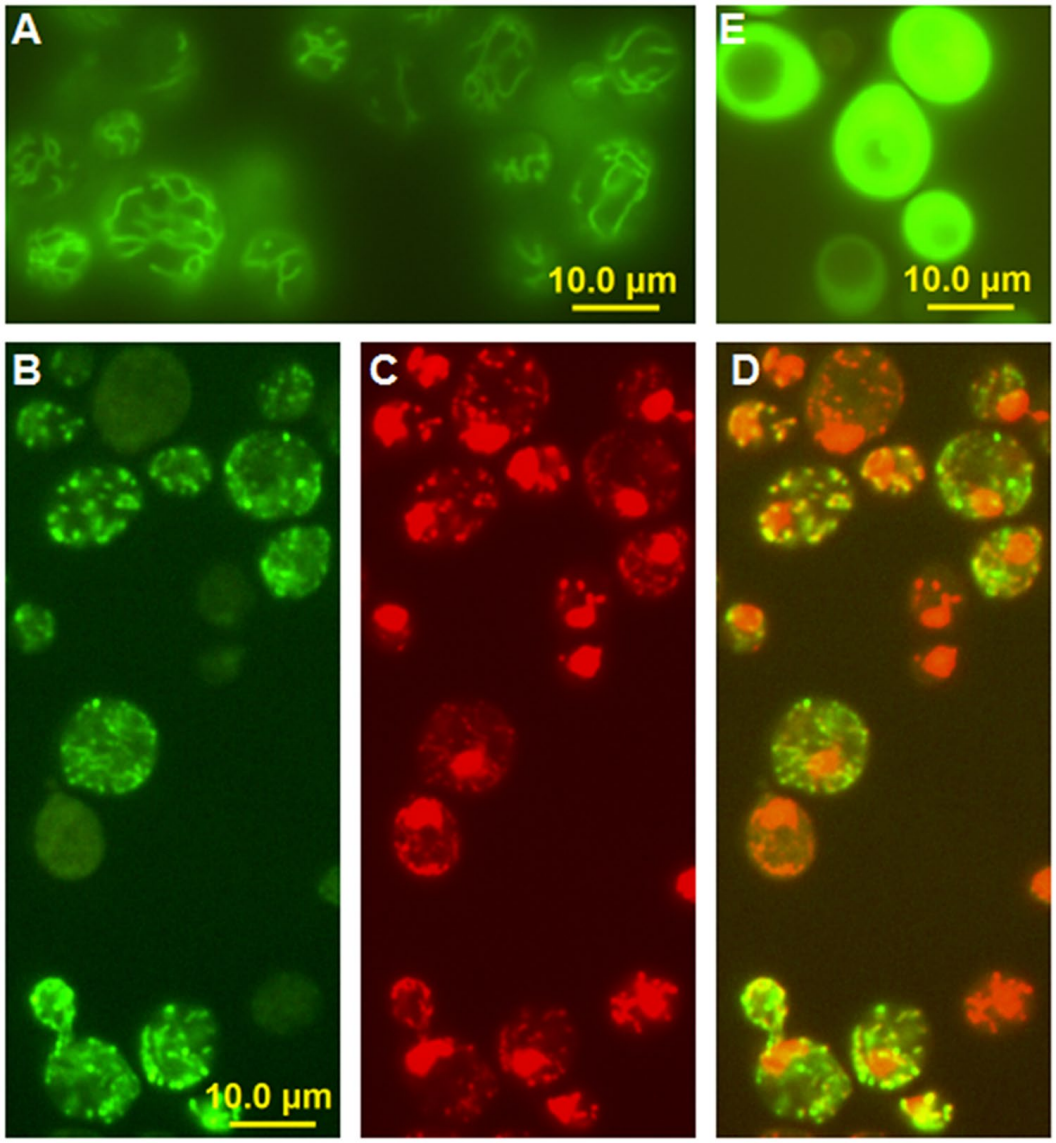
Fluorescence microscopy showing the mitochondrial localization of yeast Lon protease. (**A**) The mitochondrial network in *Saccharomyces cerevisiae* cells after DiOC_6_ staining. (**B**) The localization of Lon-GFP expressed from pUG35 in *S. cerevisiae*. (**C**) Visualization of *S. cerevisiae* nuclear (large red dots) and mitochondrial DNA (smaller red spots) after DAPI staining. The blue colour of DAPI was changed to red in the imaging software. (**D**) The merged signal confirming the co-localization of Lon-GFP and mtDNA. (**E**) A negative control, showing the non-specific GFP signal from an empty pUG35 plasmid which localizes to the cytosol of *S. cerevisiae* cells.

Human Lon protease has been clearly shown to localize to the mitochondria and to be part of the mitochondrial nucleoid^11, 30–33^. To confirm the localization of yeast Lon to the mitochondrial nucleoid, we expressed a GFP-fused Lon in *Saccharomyces cerevisiae* BY4742 cells. The *S. cerevisiae* mitochondrial network was visualized by fluorescent DiOC_6_ staining (Thermo Scientific) (Fig. 1A). The Lon-GFP fluorescence signals appeared as discrete foci within the yeast cells (Fig. 1B), and overlapped with the DNA-specific DAPI-stained mitochondrial DNA (smaller red spots in Fig. 1C,D). This coincidence of signals confirms that the mitochondrial localization of the yeast Lon protease is similar to the previously reported mitochondrial localization of its human homologue.

### Purification and activity assays of potential yeast Lon protease substrates

To study the ability of Lon to degrade mitochondrial nucleoid-associated proteins in *Saccharomyces cerevisiae*, we cloned and expressed a recombinant gene for the yeast mitochondrial mtDNA packaging protein Abf2 lacking the N-terminal mitochondrial-targeting sequence and fused with a hexahistidine tag (6 × His) using the pOPINJ expression system in *E. coli*. Native Abf2 was obtained by treating the purified recombinant protein with PreScission protease to remove the N-terminal GST affinity tag; the shortened protein was subsequently isolated on a heparin column. A final purification step by size-exclusion chromatography confirmed that Abf2 forms a dimer in solution, similar to TFAM (Supplementary Figs S1 and S2).

**Figure 2:**
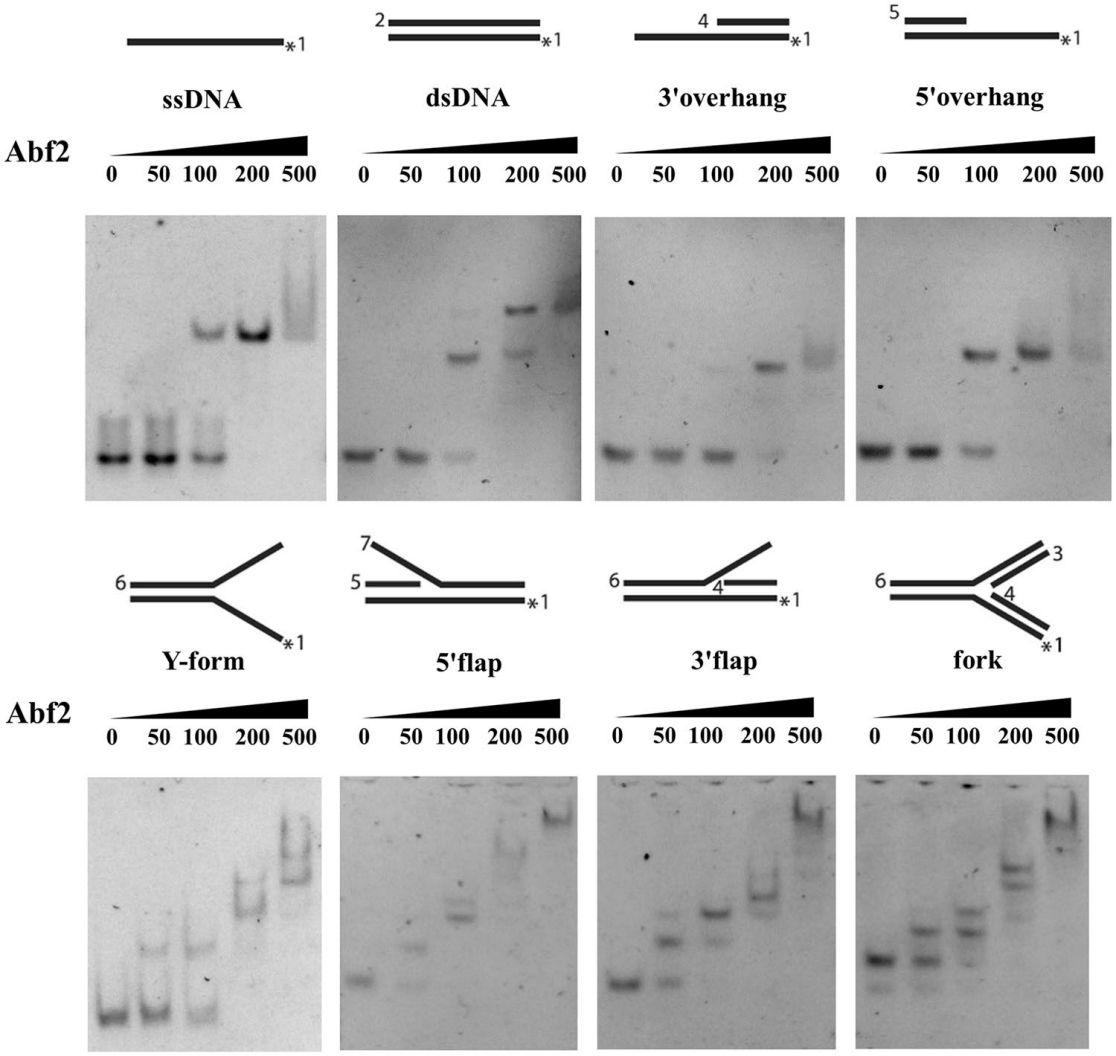
EMSA of Abf2 with various DNA substrates. Increasing concentrations of Abf2 (0, 50, 100, 200 and 500 nM) were incubated with 75 nM fluorescently labelled DNA probes, whose structures are shown schematically above the corresponding lines. The asterisk (*) indicates the position of the Cy3 fluorescent dye. Samples were separated in 8% native polyacrylamide gels in 0.5 × TBE.

Electrophoretic mobility gel shift assays (EMSA) with fluorescently labelled DNA probes of various shapes (Fig. 2 and Supplementary Table S4) were used to test the DNA binding activity of Abf2 and to observe whether it shows any preference for a particular DNA structure. Six different fluorescent probes, 3′ overhang, 5′ overhang, Y-form, 3′ flap, 5′ flap and fork, were tested with increasing concentrations of protein and the resulting gel shifts were compared to those observed with ssDNA and dsDNA (Fig. 2). Abf2 actively bound all tested DNA structures, with a slightly higher preference for dsDNA and probes having at least one free end (i.e. the 5′ overhang, 5′ flap and 3′ flap).

Abf2 DNA-binding properties were more closely observed by comparing its tendency to bind ssDNA and dsDNA. The results presented in Fig. 2 show higher affinity for dsDNA than ssDNA, supported by the comparative EMSA assay shown in Supplementary Fig. S3. Both experiments confirmed that Abf2 associates with both types of DNA, as found previously^34, 35^.

In addition to Abf2, we also studied another *S. cerevisiae* nucleoid-associated protein, the mitochondrial genome maintenance protein Mgm101. As for Abf2, we cloned and expressed a recombinant gene encoding Mgm101 without an N-terminal mitochondrial-targeting sequence fused to a 6 × His tag using a pET100/D-TOPO expression system in *E. coli*. Mgm101 was obtained by affinity purification and gel filtration, and the activity of the isolated protein was then determined by its DNA-binding ability, according to Pevala *et al*.^28^ (Supplementary Fig. S4).

### Purification and activity assays of potential human Lon protease substrates

To identify human mitochondrial nucleoid proteins that might be substrates for Lon protease, the gene corresponding to the human mitochondrial helicase Twinkle, lacking the mitochondrial targeting sequence, was cloned into a plasmid containing a C-terminal 6 × His-Halo7 purification tag and expressed in *E. coli*. This His-tagged form of Twinkle was isolated on an affinity column and further purified by size-exclusion chromatography, followed by removal of the tag with PreScission protease (Supplementary Fig. S5). The ATPase and helicase activities of the protein were then tested. The ATPase activity was measured as the amount of inorganic phosphate released in a set time using a modified form of the Lanzetta *et al*.^36^ method. Helicase activity was based on Twinkle’s ability to unwind a fluorescently-labelled dsDNA substrate with increasing concentration of enzyme added to the reaction mixture (Supplementary Fig. S6). Both assays confirmed that purified Twinkle is a functional helicase.

**Figure 3:**
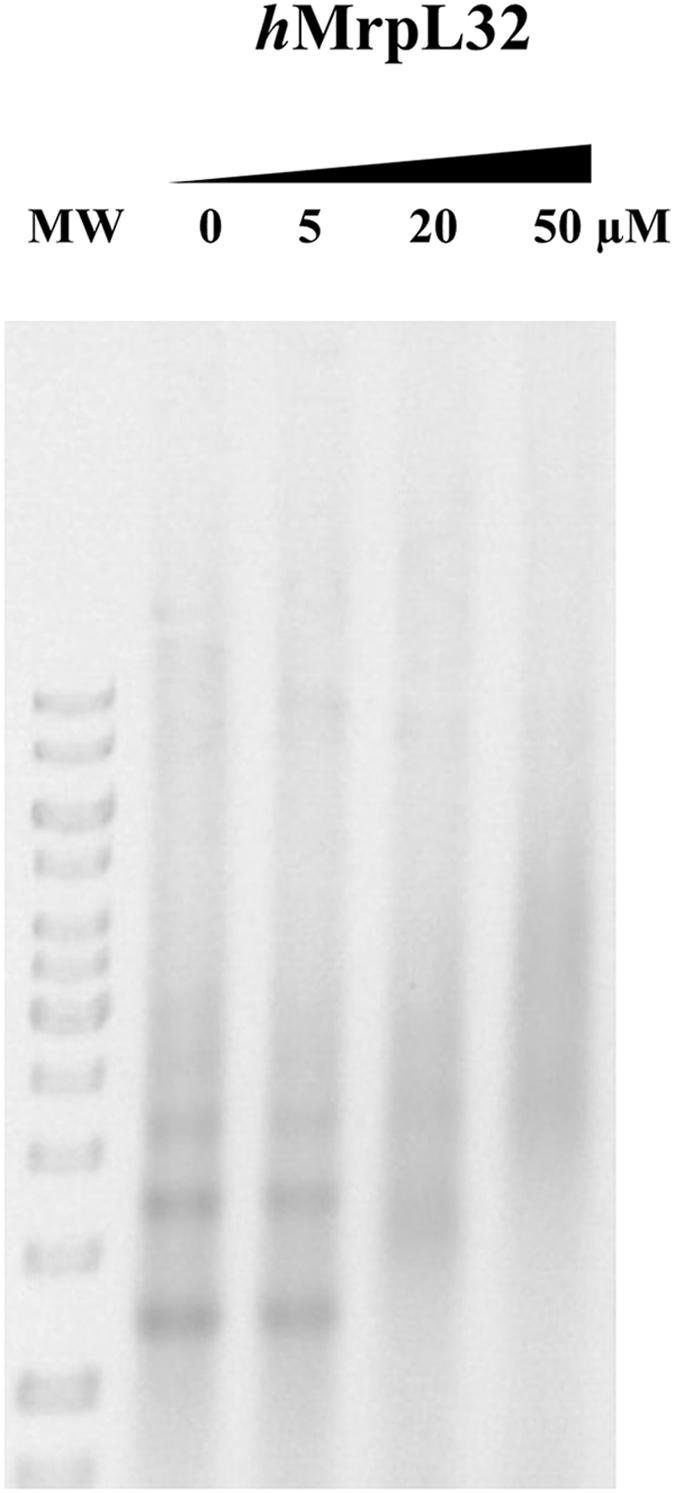
EMSA of the human large mitochondrial ribosomal subunit MrpL32 with RNA. Increasing concentrations of MrpL32 (0, 5, 20, and 50 µM) were incubated with 2 µg total bacterial RNA from *Streptomyces coelicolor*. Samples were separated in a 0.7% native agarose gel in 0.5 × TBE.

The gene encoding the human large ribosomal subunit MrpL32 was similarly cloned into a pOPINTRX vector and expressed in *E. coli*. MrpL32 was obtained by isolating the tagged version by affinity chromatography, subsequently removing the N-terminal thioredoxin tag, and purifying by size-exclusion chromatography (Supplementary Fig. S7). The activity and proper folding of this protein were determined by testing its nucleic acid binding ability using EMSA with ribosomal RNA isolated from bacterial cell culture. In this *in vitro* analysis, MrpL32 was able to actively interact with RNA (Fig. 3) indicating that the protein was functional and that this RNA substrate should be appropriate for the Lon digestion assays described later.

### The influence of potential protein substrates and DNA on *h*Lon activities

Our previous study showed that the ATPase activity of *h*Lon is stimulated in the presence of the substrate β-casein^37^. Proteins which are likely to be substrates of Lon should exhibit similar effects on its ATPase activity. We therefore tested Lon’s ATPase activity in the presence of TFAM, Abf2, Mgm101, *h*MrpL32, and BSA (a negative control). The Twinkle helicase was not included in this set because its own ATPase activity would have interfered with the measurement (Supplementary Fig. S6). The ATPase activity of *h*Lon was determined using the colorimetric assay described earlier for the Twinkle helicase. The ATPase activity measured in the presence of the potential substrates showed the same stimulation as was observed for β-casein (Table 1, Supplementary Fig. S8), but no such stimulation occurred in the presence of the BSA negative control.

**Table 1:** *h*Lon ATPase activities (nmol PO_4_^3−^/min) in the presence of possible protein substrates.

Human Lon ATPase activity			
Substrate	Basal	Stimulated	% Basal
β-casein	0.087 ± 0.003	0.210 ± 0.010	239
Abf2	0.130 ± 0.005	0.210 ± 0.020	164
Mgm101	0.117 ± 0.006	0.250 ± 0.010	209
TFAM	0.117 ± 0.004	0.240 ± 0.010	206
MrpL32	0.122 ± 0.004	0.167 ± 0.007	137
BSA	0.123 ± 0.004	0.121 ± 0.002	98

The effects of adding ssDNA, dsDNA or RNA on the Lon ATPase, protease, and peptidase activities were also tested. The protease activities were measured by observing the fluorescence resulting from FITC-casein digestion, while the peptidase activities were determined by observing the fluorescence arising from the MNA dye released by cleaving the GAAF-MNA fluorogenic peptide. None of the nucleic acids were observed to influence any of these three activities, either by themselves or in the presence of a natural *h*Lon substrate (TFAM) (Supplementary Figs S9, S10 and S11).

### Substrate digestion by Lon and the effect of nucleic acid binding

**Figure 4:**
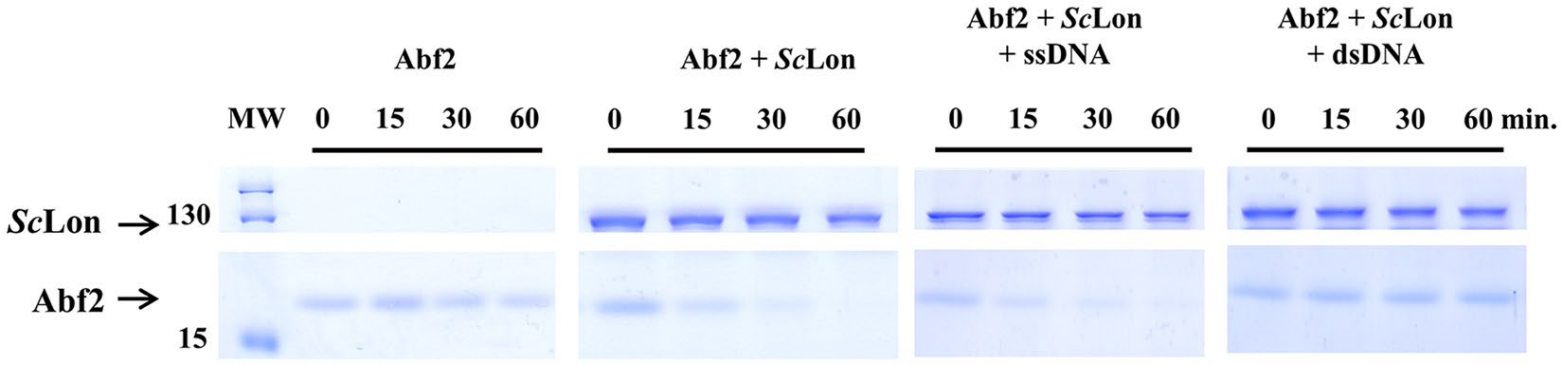
The course of Abf2 digestion by *Sc*Lon. 1.3 μg Abf2 were incubated with 2.4 µg *Sc*Lon in the presence or absence of a DNA probe in reaction mixtures containing 2 mM ATP and 10 mM MgCl_2_. Samples were withdrawn at the indicated times and loaded on a 12% SDS-polyacrylamide gel. MW – molecular weight marker.

A previous study on the regulation of TFAM levels by DNA and by phosphorylation in its HMG1 box showed that only DNA-free TFAM is a substrate for human mitochondrial Lon^9^. Our control experiments confirmed both results: TFAM free of DNA was actively digested by *h*Lon and remained untouched when bound to either single- or double-stranded DNA (Supplementary Fig. S12). Digestion of Abf2, the analogous mtDNA-packaging protein from *S. cerevisiae* by *Sc*Lon was tested in the presence and absence of DNA. Purified Abf2 and *Sc*Lon were incubated in the presence of ATP and Mg^2+^ to confirm that Abf2 is degraded by *Sc*Lon (Fig. 4). Abf2 was also incubated with either ssDNA or a linear dsDNA fragment and *Sc*Lon degradation was then assayed. Because Abf2 is known to effectively bind mtDNA at various positions no effort was made to ensure that these fragments corresponded to any particular mtDNA sequence. The results showed that, like TFAM, Abf2 is also degraded by a Lon protease in the absence of DNA. dsDNA-bound Abf2 was also not degraded by *Sc*Lon, but ssDNA binding did not appear to protect Abf2 from digestion (Fig. 4, Supplementary Fig. S13A).

Since Lon is known to bind ssDNA^38, 39^, a control experiment was carried out in which *Sc*Lon digested β-casein under the same conditions as Abf2. The course of β-casein digestion by *Sc*Lon was unaffected by the presence of either ssDNA or dsDNA, which is consistent with the above results indicating that DNA has no influence on *Sc*Lon’s protease activity (Supplementary Fig. S14).

**Figure 5:**
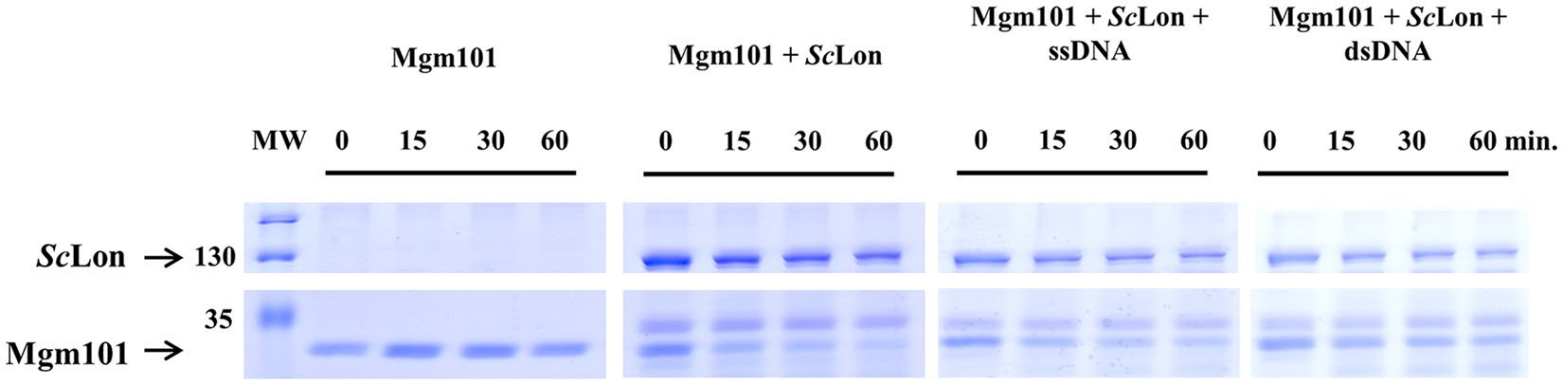
Mgm101 digestion by *Sc*Lon. 1 μg Mgm101 was incubated with 2.4 µg *Sc*Lon in the presence or absence of a DNA probe in reaction mixtures containing 2 mM ATP and 10 mM MgCl_2_. Samples were withdrawn at the indicated times and loaded on a 12% SDS-polyacrylamide gel. MW – molecular weight marker.

**Figure 6:**
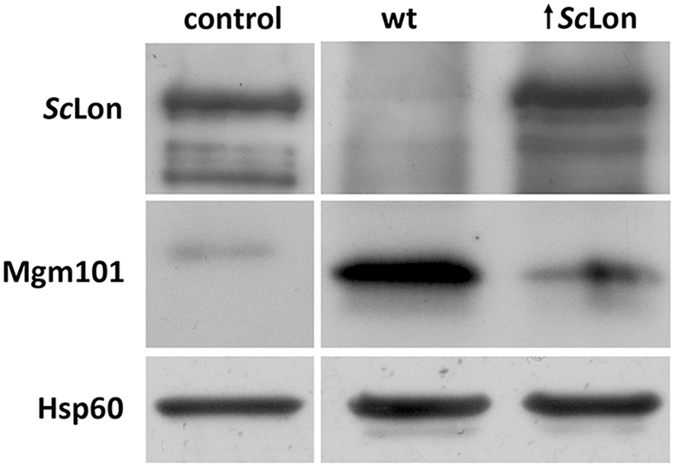
Mgm101 immunoblot analysis in *S. cerevisiae* mitochondria. 25 µg of total yeast mitochondrial proteins from *S. cerevisiae* were used for Mgm101 immunoblot detection in wild type (wt) and *Sc*Lon-overexpressing (↑ *Sc*Lon) in mitochondria. Yeast mitochondrial Hsp60 (60 kDa)^67^ was used as the protein amount reference. A 120-kDa His-tagged *S. cerevisiae* Lon protein was used as a yeast Lon protease standard. As an Mgm101 standard, a 31.7-kDa His-tagged version of Mgm101 was used in comparison with its 27.6-kDa native version.

We employed a similar approach to study another *S. cerevisiae* nucleoid-associated protein, the mitochondrial genome maintenance protein Mgm101. *Sc*Lon-mediated proteolysis was again examined in the presence or absence of DNA, and we again observed the active degradation of the protein (Fig. 5, Supplementary Fig. S13B). This time, both DNA probes appeared to partially protect the substrate, with dsDNA more strongly blocking the *Sc*Lon proteolytic activity than ssDNA, but the effect was much weaker than for Abf2. Here, we also performed an *in vivo* analysis of Mgm101 presence in *S. cerevisiae* mitochondria in various Lon-associated genetic backgrounds, which provided clear evidence that Mgm101 levels are decreased in *Sc*Lon protease-overproducing strain (Fig. 6).

**Figure 7:**
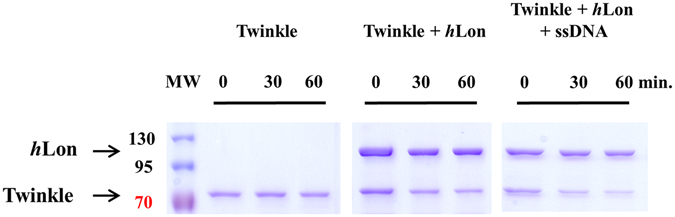
Twinkle helicase digestion by *h*Lon. 0.5 µg Twinkle helicase was incubated with 1 µg *h*Lon in the presence or absence of a ssDNA probe in reaction mixtures containing 2 mM ATP and 10 mM MgCl_2_. Samples were withdrawn at the indicated times and loaded on a 12% SDS-polyacrylamide gel. MW – molecular weight marker.

Moving on to the human nucleoid-associated proteins, Twinkle helicase, a core nucleoid protein, was degraded by *h*Lon when no DNA was present (Fig. 7). Interestingly, Twinkle was degraded by *h*Lon even in the presence of both ssDNA (Fig. 7, Supplementary Fig. S13C) and dsDNA with either 3′ or 5′ overhangs (Supplementary Fig. S15). This was somewhat unexpected given that every other DNA-binding protein we examined here was at least partially protected by DNA binding.

**Figure 8:**
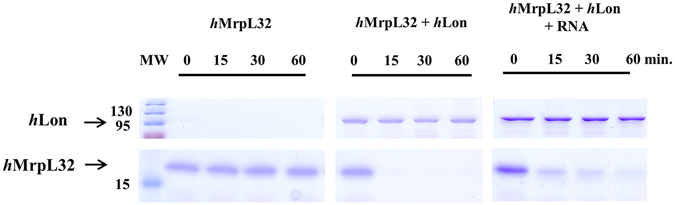
MrpL32 digestion by *h*Lon. 2 µg MrpL32 was incubated with 0.5 µg *h*Lon in the presence of an RNA probe in reaction mixtures containing 2 mM ATP and 10 mM MgCl_2_. Samples were withdrawn at the indicated times and loaded on a 15% SDS-polyacrylamide gel. MW – molecular weight marker.

Human Lon protease was earlier found to bind RNA as well as DNA^38^, and *in vivo* experiments with *Sc*Lon indicated that it degrades some ribosomal proteins^12^. To examine the possible function of *h*Lon in ribosome dynamics, we tested the *h*Lon-mediated digestion of the human large ribosomal subunit protein MrpL32. Previously, *y*MrpL32 was shown to be proteolytically cleaved by the inner mitochondrial membrane protease *m*-AAA, which is responsible for its maturation and the assembly of mitochondrial ribosomes^40^. Bonn *et al*.^41^ further found that this processing is not dependent on particular cleavage sequences but rather on the actual shape of the protein. No information concerning the behaviour of human MrpL32 within human mitochondria is currently available, however. By analogy with the yeast MrpL32, *h*MrpL32 might be expected to be a *h*Lon substrate, and indeed purified human MrpL32 was very rapidly digested by *h*Lon (in less than 15 minutes). Digestion of MrpL32 was somewhat slowed when it was pre-incubated with RNA prior to digestion, (Fig. 8, Supplementary Fig. S13D) showing that RNA binding slightly protected MrpL32 against degradation.

## Discussion

In this study, we examined the ability of the ATP-dependent mitochondrial matrix protein Lon to degrade four mitochondrial proteins involved in, or expected to be involved in, mtDNA packaging (Abf2) and recombination (Mgm101) in yeast and mtDNA replication (Twinkle) and translation (MrpL32) in humans. We also studied human TFAM both to verify previous work on this protein^9^ and to more accurately compare it with Abf2. We verified the correct folding of each protein by conducting DNA-binding EMSA studies and functional assays, and we examined the ability of Lon to digest these proteins alone or when bound to nucleic acids. The *in vitro* digestion of Mgm101 was confirmed *in vivo* by an immunoblot analysis of the level of Mgm101 in *Sc*Lon-overproducing cells compared to wild-type cells (Fig. 6). We also tested the ability of each protein to stimulate Lon’s ATPase activity, a behaviour exerted by all known and characterized Lon substrates. A prerequisite for inferring that the results of these assays reflect events in the mitochondria is the verification that Lon does, in fact, localize to the mitochondria in these two organisms. Human Lon protease has been clearly shown to localize to the mitochondria and to be part of the mitochondrial nucleoid^11, 30–33^; however, the localization of *S. cerevisiae* Lon has not previously been clearly demonstrated *in vivo*. We here show that a yeast Lon does, in fact, localize with the mitochondrial nucleoid using a combination of GFP fluorescence to identify the positions of Lon and DAPI staining to locate the cellular DNA (Fig. 1d).

Lon was originally observed to degrade oxidatively damaged, partially unfolded, and short-lived regulatory proteins^42^. More recently, the ability of Lon to regulate the levels of proteins responsible for various mitochondrial processes has been studied. The most through of these has been the exploration of the effect of the Lon proteolysis on the mtDNA packaging protein TFAM. In particular, it has recently been shown that proteolysis of TFAM by Lon is one part of a mechanism that rapidly modulates the levels of TFAM, and consequently of mtDNA, in mitochondria^9^. The yeast counterpart of TFAM is Abf2. Although this protein has clearly been shown to be the yeast mtDNA packaging protein^43^, it has not yet been shown to play a role in mtDNA replication and gene expression, which TFAM does^7^. Consequently, the role and the regulation of Abf2 in yeast might be different from the role and regulation of TFAM in humans. To address this question, we studied the DNA-binding and Lon digestion profiles of both proteins under identical conditions. We found that Abf2 actively binds a wide variety of DNA structures with apparently higher affinity for DNA probes resembling DNA recombination intermediates (i.e. the 5′ overhang and the 3′ and 5′ flaps). These observations are consistent with those of Diffley and Stillman^35^ who found that Abf2 showed comparable binding affinity for most DNA fragments except those possessing simple poly A sequences. Like TFAM, binding to dsDNA protected Abf2 from degradation by Lon (Fig. 4), but unlike it, binding to ssDNA provided little to no protection. Since binding to the single-stranded transcription promoter is an important part of TFAM’s function, it is perhaps not unexpected that TFAM bound to ssDNA would resist Lon proteolysis in the same way that it does when bound to dsDNA. That Abf2 appears to bind more weakly to ssDNA than to dsDNA and is not protected from Lon by doing so suggests that Abf2 is not likely to serve as a transcriptional activator in the same way as TFAM.

Disruption of the *ABF2* gene *in vivo* causes severe disintegration of mtDNA, which is noticeably less protected and exhibits increased sensitivity to nuclease attack and chemical damage by oxidative stress^44, 45^. These effects can be eliminated by the presence of other HMG proteins, including the yeast nuclear protein NHP6A, the human TFAM, and even the *E. coli* HU protein, when targeted to the yeast mitochondria^46, 47^. At least two of these proteins, TFAM and HU, are known to be degraded by their respective organism’s Lon protease. Together with our demonstration that Abf2 is also a Lon substrate, these findings suggest that the ability of Lon to regulate the levels of mtDNA packaging proteins may be a general characteristic.

For the remaining three proteins, we found that Mgm101 and MrpL32 were only partially protected when bound to DNA (either ss or ds) and RNA, respectively, while DNA binding appeared to offer no protection at all to the Twinkle helicase. In every case, it is possible to infer plausible mechanistic reasons why the given protein is completely, partially, or not at all protected from Lon digestion by nucleic acid binding. Lon prefers to cleave between hydrophobic residues which are normally buried in the core of folded proteins or within the interface of protein complexes but which become exposed either by unfolding or complex disassembly^22, 48^; such regions spatially adjacent to charged areas seem to be particularly favoured^22^.

It was previously suggested that Lon may target the HMG boxes in TFAM: they contain a cluster of conserved hydrophobic residues which are shielded from solvent in the DNA complex, but may be exposed in unbound TFAM^4^. The TFAM–dsDNA crystal structure is consistent with this hypothesis^49^, and it shows, moreover, that these residues are also flanked by a number of positively-charged residues, making this location appear likely to be a favoured Lon cleavage site (Supplementary Fig. S16). A homology model of Abf2 based on the TFAM–dsDNA structure prepared using the Phyre2 server^50^ shows that all these residues are largely conserved in the HMG box of Abf2 (Supplementary Fig. S16), suggesting a conserved cleavage site between TFAM and Abf2, and one presumably shared with all HMG box proteins.

Recently, the 3.4 Å electron microscopy structure of the human mitochondrial ribosome was reported, which includes the entire mature form of MrpL32 (residues 79–188)^51^. In this structure, the N-terminal 49 residues are buried within the ribosome itself, leaving much of the rest of the proteins surface exposed. Of these 49, 23 are deeply buried in the 16S rRNA while the remaining 26 are sequestered by the MrpL3, MrpL17, MrpL22, and MrpL45 ribosomal proteins, which form a layer atop the 16S rRNA. This organization immediately suggests why RNA binding only partially protected MrpL32 from Lon, only part of the normally sequestered area was bound. It is worth noting that the area normally in contact with the ribosomal proteins lacks secondary structure (apart from an isolated β-bridge), and does contain a number of hydrophobic residues, making it a plausible location for a Lon cleavage site.

Although no high-resolution structures are available for Mgm101 and the Twinkle helicase, their known biochemical characteristics can still be used to infer likely reasons for their Lon degradation profiles. The N-terminal region of Mgm101 appears to be intrinsically disordered, making it a possible Lon-recognition region; this region is thought to be important for the recognition of other mitochondrial proteins^52^. Whether this region is involved in DNA binding is not clear: there is clear evidence that part of the C-terminal region is involved^27^, that there is some sort of large-scale structural change when DNA binds^10^, and that the N-terminus is necessary for the formation of the large-scale structure of Mgm101^10^, but it has not been shown either that the N-terminus binds DNA or what, exactly, the role of the N-terminus is in forming the large-scale structure. Mgm101 appears to be involved in recombination-mediated DNA repair^53^, presumably in concert with other proteins. The proteins which make up this recombination complex are still unknown, but since Mgm101 cannot catalyse strand recombination by itself, they must exist. Consequently, Mgm101, like MrpL32, exerts its biological function in a complex containing both proteins and nucleic acids, meaning that DNA binding alone is presumably not enough to shield all potential Mgm101 Lon target sites.

Finally, the Twinkle helicase is known to be one of the proteins necessary for mtDNA replication, though the actual details of this process remain unknown^26, 54^. A low-resolution electron microscopy structure of Twinkle shows that it has a two-layer hexameric structure, with a fairly well-ordered layer formed by interactions between the C-terminal helicase domains and a more variable layer formed by the N-terminal pseudo-primase domains^55^; these two domains appear to be connected by a flexible linker. If Twinkle works like other hexameric helicases^56^, then it would be expected to operate as part of a larger mtDNA replication complex; the DNA binding site, normally through the center of the hexamer, would also be expected to be separate and distinct from the part associated with other replisome proteins. Structures of hexameric replicative helicases bound to DNA (e.g. DnaC^57^) suggest that DNA binding does not change the conformation of the helicase in such a way that previously exposed regions become protected from Lon recognition.

Taking all of this together, we see that the level of protection DNA or RNA binding offers a given substrate is related to how closely the resulting protein–nucleic acid complex is to the protein’s putatively complete functional complex. The more complete the likely functional form, the greater the degree of protection to the constituent protein. Thus TFAM and Abf2, which are in their complete functional forms when DNA-bound, are the most protected, Mgm101 and MrpL32, which are in partially assembled forms, are only partially protected, and Twinkle, which is active but outside the context of a replisome, is not protected at all. In these four cases, Lon can be seen exercising its previously known function of degrading the individual components of non-assembled protein complexes. These four proteins are all involved in mtDNA replication (and therefore indirectly in gene expression), repair and recombination, and translation, making the Lon protease an important tool for regulating these processes in mitochondria.

To summarize, we have shown that a yeast Lon localizes to the *S. cerevisiae* mitochondrial nucleoid and have identified *in vitro* four new substrates for the Lon protease, two each from *S. cerevisiae* and humans. We have shown that yeast Abf2, though it has some differences from human TFAM, is degraded by Lon under many of the same conditions, suggesting that the regulatory model for TFAM might also be applicable to Abf2. We have also found that yeast Mgm101 and human MrpL32 are degraded by Lon, but are partially protected by binding, respectively, DNA and RNA, suggesting that the DNA and RNA binding regions of these proteins are adjacent to those regions where they associate with other proteins in their functional complexes. Finally, we found that the human Twinkle helicase is not protected at all by DNA binding. Since all four of these proteins are involved in mtDNA replication, repair, recombination, translation, or packaging, we have shown that the Lon protease is likely to play an important role in the regulation of all of these processes, and therefore also in nucleoid dynamics.

## Methods

### Expression and purification of recombinant proteins

#### Microbial strains and growth media

*Escherichia coli* TG1 (Stratagene) was used to amplify plasmid constructs and Rosetta 2 (DE3) cells (Merck) were used to produce the recombinant proteins Abf2, Mgm101, TFAM, Twinkle helicase, *h*MrpL32, and *h*Lon. *E. coli* TG1 cultures were grown at 37 °C in LB medium (1% (w/v) bactotryptone, 0.5% (w/v) yeast extract, 1% (w/v) NaCl, pH 7.5) containing 100 μg/ml ampicillin. *E. coli* Rosetta 2 (DE3) cultures were grown at 37 °C in TB medium (1.2% (w/v) bactotryptone, 2.4% (w/v) yeast extract, 72 mM K_2_HPO_4_, 17 mM KH_2_PO_4_, 0.4% (w/v) glycerol) containing 0.5% (v/v) glucose with 100 μg/ml ampicillin and 34 μg/ml chloramphenicol.

#### Expression and purification of Abf2

Recombinant *S. cerevisiae* Abf2 lacking the mitochondrial targeting sequence (first 26 residues) was prepared by in-fusion cloning (Clontech) into a pOPINJ vector carrying a double N-terminal glutathione and hexahistidine tag (6 × His-GST)^58^. A PCR amplification was carried out using the Abf2 pOPINJ FW and Abf2 pOPINJ RV primers (Supplementary Table S1). All plasmid constructs used in this study may be found in Supplementary Table S2. This construct was expressed in *E. coli* Rosetta 2 (DE3) cells induced with 20 µM IPTG overnight at 18 °C. Cells were harvested and resuspended in buffer A (50 mM ammonium acetate, pH 7.3, 1 M KCl, 10% (v/v) glycerol) with the addition of 0.1% Tween 20 and 0.2 mg/ml lysozyme followed by a 15-minute room temperature incubation and sonication on ice. The cell lysate was centrifuged for 20 minutes at 100,000 × g and the supernatant was loaded onto a Ni Sepharose 6 Fast Flow column (GE Healthcare). The column was washed with five column volumes of solubilization buffer A containing 40 mM imidazole and five column volumes of buffer B (50 mM ammonium acetate, pH 7.3, 500 mM KCl, 10% (v/v) glycerol) containing 40 mM imidazole. The protein bound to the column was then eluted in buffer B with 0.5 M imidazole. Elutions containing Abf2 were pooled and the 6 × His-GST tag was removed by overnight incubation with PreScission protease at 6 °C following the GE Healthcare protocol and then applied onto a Glutathione Sepharose 4 Fast Flow column (GE Healthcare) equilibrated in buffer B. After a 30-minute incubation, Abf2 was eluted in the unbound fraction, diluted 2.5 × with a buffer B lacking KCl, and loaded onto a Heparin Sepharose 6 Fast Flow column (GE Healthcare). The bound protein was eluted with a stepwise KCl gradient (0.6, 0.8, 1 and 1.5 M), concentrated and purified on a Superdex 75 10/300 GL column (GE Healthcare) in buffer B with 5% (v/v) glycerol.

#### Expression and purification of Mgm101

Recombinant *S. cerevisiae* Mgm101 without the mitochondrial targeting sequence and carrying an N-terminal 6 × His tag was cloned into a pET100/D-TOPO (Invitrogen) vector and expressed in *E. coli* Rosetta 2 (DE3) cells as described in Pevala *et al*.^28^.

#### Expression and purification of *Sc*Lon

Yeast Lon carrying a C-terminal 6 × His tag was overproduced and purified from isolated yeast mitochondria as described previously^59^. In brief, mitochondria at 10 mg/ml were resuspended in buffer C (20 mM HEPES, pH 8.0, 150 mM NaCl, 5 mM MgCl_2_, 20% (v/v) glycerol) containing 1 mM ATP and 1.6 mg of Lubrol/mg of mitochondrial protein. The lysate was centrifuged at 200,000 × g at 4 °C for 10 minutes and the supernatant was applied to a Ni Sepharose 6 Fast Flow column (GE Healthcare). The column was washed with five column volumes of buffer C containing 40 mM imidazole, and bound protein was then stepwise eluted with buffer C containing 0.1, 0.2 and 0.3 M imidazole. The enzymatic activity of the enzyme was then determined by FITC-casein degradation as described in Ambro *et al*.^37^.

#### Expression and purification of TFAM

Human recombinant TFAM lacking the mitochondrial targeting sequence (the first 30 amino acids) was cloned into a pET22 (b+) vector carrying a C-terminal 6 × His tag (gift of CK. Suzuki) and expressed in *E. coli* Rosetta 2 (DE3) cells induced with 0.5 mM IPTG for 2 h at 37 °C. Cells were harvested and resuspended in buffer D (20 mM HEPES, pH 8.0, 500 mM NaCl, 20% (v/v) glycerol) and sonicated on ice. The cell lysate was centrifuged for 30 minutes at 100,000 × g and the supernatant was loaded onto a Ni Sepharose 6 Fast Flow column (GE Healthcare). The column was washed with five column volumes of buffer D containing 40 mM imidazole, and protein bound to the column was then eluted in buffer D with a stepwise imidazole gradient (0.1, 0.2, 0.3 and 0.5 M). Elutions containing TFAM were pooled, concentrated, and applied to a Superdex 200 Increase 10/300 GL column (GE Healthcare) in buffer E (25 mM HEPES, pH 8.0, 1 M NaCl, 5% (v/v) glycerol). Before the Lon digestion experiments, this TFAM was desalted by filtration into buffer E, containing only 150 mM NaCl.

#### Expression and purification of Twinkle helicase

Recombinant human Twinkle helicase lacking the mitochondrial targeting sequence (the first 42 residues) was cloned into a pOPIN-3C-HALO7 vector carrying a C-terminal 6 × His-Halo7 tag^58^. PCR amplification was carried out using the primers Peo1 pOPIN FW and Peo1 pOPIN RV. The construct was expressed in *E. coli* Rosetta 2 (DE3) cells induced with 20 µM IPTG overnight at 18 °C. Cells were harvested and resuspended in buffer F (50 mM Tris-HCl, pH 7.4, 1 M KCl, 10% (v/v) glycerol, 10 mM imidazole, 5 mM β-mercaptoethanol, 0.1% Igepal CA-630) with the addition of 0.2 mg/ml lysozyme and sonicated on ice. The cell lysate was centrifuged for 30 minutes at 100,000 × g and the supernatant was loaded onto a Ni Sepharose 6 Fast Flow column (GE Healthcare). The column was washed with five column volumes of buffer F containing 40 mM imidazole and the same volume of buffer G (50 mM Tris-HCl, pH 7.4, 500 mM KCl, 10% (v/v) glycerol, 5 mM β-mercaptoethanol) containing 40 mM imidazole. Protein bound to the column was then eluted in buffer G with a stepwise imidazole gradient (0.1, 0.2, 0.3 and 0.5 M). Elutions containing Twinkle were pooled, concentrated and applied to a Superose 6 10/300 GL column (GE Healthcare) in buffer G with 5% (v/v) glycerol. The fractions containing Twinkle were cleaved by overnight incubation with PreScission protease at 6 °C according to the GE Healthcare protocol and subsequently re-applied to the same Ni Sepharose 6 Fast Flow column (GE Healthcare) and purified accordingly. Optionally, the cleaved, un-tagged Twinkle was further purified on a Heparin Sepharose 6 Fast Flow column (GE Healthcare) equilibrated in buffer H (50 mM Tris-HCl, pH 7.4, 150 mM KCl, 150 mM imidazole, 5% (v/v) glycerol). The bound protein was then eluted with a stepwise KCl gradient (0.4, 0.6, 0.8, 1 and 1.5 M). The functionality of this helicase was tested by measuring its ATPase and helicase activities.

#### Expression and purification of *h*MrpL32

Human ribosomal subunit MrpL32 lacking the mitochondrial targeting sequence (the first 39 residues) was cloned into a pOPINTRX vector carrying an N-terminal 6 × His–thioredoxin tag^58^ and expressed in *E. coli* Rosetta 2 (DE3) cells induced with 20 µM IPTG overnight at 18 °C. The cells were harvested and resuspended in buffer J (25 mM HEPES pH 7.4, 1 M NaCl, 10% glycerol, 0.1% Igepal CA-630, 1 mM DTT) with the addition of 0.2 mg/ml lysozyme and sonicated on ice. The cell lysate was centrifuged for 15 minutes at 100,000 × g and the supernatant was loaded onto a cOmplete His-tag purification resin (Roche). The column was washed with five column volumes of buffer J followed by five volumes of buffer K (25 mM HEPES pH 7.4, 500 mM NaCl, 10% glycerol, 0.1% Igepal CA-630, 1 mM DTT). The protein bound to the column was then eluted in buffer K with a stepwise imidazole gradient (0.05, 0.1, 0.15 and 0.2 M). Elutions containing *h*MrpL32 were pooled, concentrated (using a 10 K MW cut-off) and applied to a Superdex 200 Increase 10/300 GL column (GE Healthcare) in buffer K with 5% (v/v) glycerol and 10 mM β-mercaptoethanol. Fractions containing MrpL32 were cleaved by overnight incubation with PreScission protease at 6 °C following the GE Healthcare protocol. After cleavage, purification on a cOmplete His-tag resin column (Roche) was repeated. Fractions containing *h*MrpL32 were pooled and protein concentration was determined by absorbance at 280 nm using a Nanodrop 2000 (Thermo Scientific). This method was preferred to the bicinchoninic acid assay used for the other proteins because the β-mercaptoethanol in the buffer interferes with the dye.

#### Isolation of human Lon

Human Lon was purified and its ATPase, peptidase and protease activities were measured as described in Ambro *et al*.^37^.

#### Purity analysis and determination of protein concentration

All purified proteins were concentrated on a Microsep Advance Centrifugal Device (10 K, 30 K, and 100 K MW cut-offs) (Pall) and analysed by SDS-PAGE and Coomassie Brilliant Blue R250 staining. The protein concentration for all but MrpL32 (see above) was determined using the Pierce bicinchoninic acid assay.

### Protein degradation assays

#### *Sc*Lon digestion of substrates

The given amounts of substrate proteins were incubated in reaction buffer (50 mM Tris-HCl, pH 8.5, 10 mM MgCl_2_, 2 mM ATP) under various conditions. A 72-bp oligonucleotide was used as the ssDNA probe (10 µM) and a 750-bp DNA fragment (KpnI/HindIII) from the pOPINM-XRCC6BP1 plasmid construct (4 µM) was used as the dsDNA probe. Each reaction was carried out at 37 °C for 60 minutes with aliquots withdrawn at the stated times. Reaction products were analysed by SDS-PAGE.

#### *h*Lon digestion of substrates

The given amounts of substrate proteins were incubated under various conditions in the reaction buffer given above, and the same ssDNA and dsDNA probes were used. For MrpL32, the total bacterial RNA (2 mg/ml) from *Streptomyces coelicolor* was used as an RNA binding probe in place of the DNA. The reactions were carried out at 37 °C for 60 minutes with aliquots withdrawn at the indicated times and the products were subsequently analysed by SDS-PAGE.

### Yeast strains growth and mitochondria isolation

The yeast synthetic media were supplemented with glucose (0.2%), galactose (1.5%) and Casamino acids (0.5%). Where required, adenine (0.002%), tryptophane (0.002%) or uracil (0.002%) was added. The yeast strains (Supplementary Table S3) were grown to an early stationary phase and mitochondria were isolated as described in Suzuki *et al*.^60^.

### Immunoblot analysis

25 µg of yeast mitochondrial proteins^61^ were separated by glycine-buffered SDS-PAGE, these proteins were transferred from polyacrylamide gel to a nitrocellulose or PVDF membrane (GE Healthcare) using a semi-dry transfer method. The membrane was then analyzed by immunoblotting with appropriate antisera (directed against *Sc*Lon, Hsp60 and Mgm101). Membrane-bound primary antibodies were detected using peroxidase-conjugated secondary antibodies (anti-rabbit IgG, Jackson ImmunoResearch), incubated with enhanced chemiluminiscence detection reagents and captured on film.

### Electrophoretic mobility shift assays (EMSA)

#### Abf2 DNA-binding assay

Oligonucleotide sequences and structures are shown in Supplementary Table S4. These oligonucleotides were modified by the Cy3 fluorescent dye at the 5′ end. All substrates were prepared as described in Matulova *et al*.^62^. The indicated amounts of Abf2 were incubated with 75 nm of each fluorescently‐labelled DNA substrates at 30 °C in 10 μl of reaction buffer (25 mM Tris-HCl, pH 7.4, 100 mM NaCl, and 0.1 mg/ml BSA) for 10 min. After the addition of gel loading buffer, the reaction mixtures were resolved in 8% native polyacrylamide gels in 0.5 × TBE buffer (45 mM Tris-HCl, 45 mM boric acid, 1 mM EDTA, pH 8.3). The gels were scanned using an Amersham Imager 600.

#### Comparative DNA-binding analysis

The indicated amounts of Abf2 or TFAM were incubated with a ssDNA (10 µM) or dsDNA (4 µM) probe at 30 °C for Abf2 or at 37 °C for TFAM in 20 μl of reaction buffer (25 mM Tris-HCl, pH 7.4, 100 mM NaCl, and 0.1 mg/ml BSA) for 10 min. The DNA probes were the same as those used in the protein digestion assays. After the addition of gel loading buffer, the reaction mixtures were resolved in 1% (w/v) agarose gel in 0.5 × TBE buffer. The gels were scanned using a BDA digital gel documentation system (Biometra).

#### MrpL32 RNA-binding assays

The given amounts of MrpL32 were incubated with a total bacterial RNA (0.8 µg/µl) probe at 37 °C in 20 μl of reaction buffer (25 mM Tris-HCl, pH 7.4, 100 mM NaCl, and 0.1 mg/ml BSA) for 10 min. After the addition of gel loading buffer, the reaction mixtures were resolved in a 0.7% (w/v) agarose gel in 1 × TAE buffer. The gels were scanned using a BDA digital gel documentation system (Biometra).

### ATPase and helicase activities of Twinkle helicase

#### ATPase activity

ATPase activity was determined as described in Ambro *et al*.^37^ using the non-radioactive colorimetric method of Lanzetta *et al*.^36^. The reaction was performed in a 300 µl volume containing 50 mM Tris-HCl, pH 8.0, 40 mM MgCl_2_, and 0.5 mM ATP at 37 °C. Aliquots were withdrawn after 0, 15 and 30 minutes and absorbance at 660 nm was measured. The activity was also determined in the presence of 4 μg denatured ssDNA. The data was analysed using R v. 3.3.0^63^.

#### Helicase activity

Twinkle helicase activity was observed by unwinding fluorescently labelled dsDNA substrates (both 5′ and 3′ overhangs) with increasing enzyme concentration. Reactions were carried out in buffer K (20 mM Tris-HCl, pH 7.5, 5 mM MgCl_2_, 4 mM DTT, 3 mM ATP, 100 µg/ml BSA) for 30 minutes at 37 °C with 75 nM dsDNA probe followed by a 5-minute incubation with Proteinase K. The reaction mixtures were resolved in 8% native polyacrylamide gels in 0.5 × TBE buffer. The gels were scanned using a KODAK Image Station 2000MM.

### Fluorescence microscopy

A pUG35-*Cp*Lon plasmid expressing full-length *Cp*Lon (CPAR2_205210) tagged with yEGFP3 at the C-terminus was constructed as follows. A pDrive-*CpLon* plasmid was digested with EcoRI and ligated into the EcoRI site of the pUG35 vector, a multi-copy vector under the control of the medium-strength *Met25* promoter^64^. The two CTG codons (244–246 and 1456–1458 bp) were mutated to TCG to preserve the same leucine when expressed in *S. cerevisiae*. The plasmid was transformed into *S. cerevisiae* BY4742 cells by electroporation^65^.

The intracellular localization of the *Cp*Lon-yEGFP3 fusion protein was determined by fluorescence microscopy according to Pevala *et al*.^66^. *S. cerevisiae* BY4742 cells transformed with pUG35-*Cp*Lon were grown at 28 °C for 36 h in SD medium with 20 µg/ml methionine and 2 µg/ml histidine, leucine, and lysine. The cell cultures were centrifuged for 10 min at 1500 × g, washed 2 × with sterile distilled water and resuspended in SD medium without methionine. After 16 h, the cells were centrifuged 10 min at 1500 × g, and washed 2 × with sterile water. Mitochondria were visualized after 15 min staining with 175 nM of the lipophilic dye DiOC_6_ (Thermo Scientific) in 10 mM HEPES pH 7.4 and 5% glucose. For DNA staining, cells were fixed with 70% ethanol (10 min), centrifuged and resuspended in 10 mM Tris-HCl pH 7.6 containing 50 ng/ml DAPI. The samples were examined with an Olympus BX-50 fluorescence microscope equipped with the corresponding filters and an OLYMPUS DP70 digital camera.

## Electronic supplementary material

Kunova et al. 2017 Supplementary Info
