# Supplementary material for: The role of Lon-mediated proteolysis in the dynamics of mitochondrial nucleic acid-protein complexes
Source: Sci Rep. 2017 Apr 4;7:631. doi: 10.1038/s41598-017-00632-8 (PMC5428876; doi:10.1038/s41598-017-00632-8)
Supplement: Supplementary file 1 — Kunova et al. 2017 Supplementary Info [file 41598_2017_632_MOESM1_ESM.pdf]

The role of Lon-mediated proteolysis in the dynamics of mitochondrial nucleic acid-protein complexes

Nina Kunová[1], Gabriela Ondrovičová[1], Jacob A. Bauer[1], Jana Bellová[1], Ľuboš Ambro[1#], Lucia Martináková[1], Veronika Kotrasová[1], Eva Kutejová[1,2*] and Vladimír Pevala[1*]

[1] Department of Biochemistry and Structural Biology, Institute of Molecular Biology, Slovak Academy of Sciences, Dúbravská cesta 21, 845 51 Bratislava, Slovakia

[2] Institute of Microbiology, Academy of Sciences of the Czech Republic, BIOCEV, Průmyslová 595, 252 42 Vestec, Czech Republic

[#] Current address: Pavol Jozef Šafárik University in Košice, Faculty of Medicine, Trieda SNP 1, 040 11 Košice, Slovakia

[*] Corresponding authors

1) Eva Kutejová
Affiliation: Department of Biochemistry and Structural Biology, Institute of Molecular Biology, Slovak Academy of Sciences, Dúbravská cesta 21, 845 51, Bratislava, Slovakia
Tel.: +421 259307442
Fax: +421 259307416
E-mail: evakutej@hotmail.com

2) Vladimír Pevala
Affiliation: Department of Biochemistry and Structural Biology, Institute of Molecular Biology, Slovak Academy of Sciences, Dúbravská cesta 21, 845 51, Bratislava, Slovakia
Tel.: +421 259307446
Fax: +421 259307416
E-mail: vladimir.pevala@savba.sk

Supplementary data:

Table S1: Primers used for PCR amplification

Primer	Sequence 5′ → 3′
Abf2_pOPINJ_FW	AAGTTCTGTTTCAGGGTACCAAGGCTTCCAAGAGAACGC
Abf2_pOPINJ_RV	CTGGTCTAGAAAGCTTTTAGTTGAGAGGGTAGCGAGC
Peo1_pOPIN_FW	AGGAGATATACCATGGAGACTCTCCAAGCCTTGG
Peo1_pOPIN_RV	CAGAACTTCCAGTTTCTTTGAACGCTTGGAGGT
MrpL32_pOPIN_FW	AAGTTCTGTTTCAGGGTACCAGTCCTCCGTGGGGACC
MrpL32_pOPIN_RV	CTGGTCTAGAAAGCTTTCAATTCTGGGTGAACCAG

Table S2: Plasmid constructs used in this study

Plasmid construct	Protein version	Host	Source
pDrive-*Cp*Lon	*Cp*Lon	*E. coli*	J. Nosek
pUG35-*Cp*Lon	*Cp*Lon-*y*EGFP3	*S. cerevisiae* BY4742	this study
pProEx1-*h*LON	*h*Lon, (Δ1–114) lacks import presequence 6×His tag	*E. coli* Rosetta 2 (DE3)	gift of C.K. Suzuki
pET22(b+)-TFAM	TFAM (Δ1–30) lacks import presequence, 6×His tag	*E. coli* Rosetta 2 (DE3)	gift of C.K. Suzuki
pET100/D-TOPO-*Sc*MGM101	*Sc*Mgm101 (Δ1–21) lacks import presequence, 6×His tag	*E. coli* Rosetta 2 (DE3)	J. Nosek[2]
pOPINJ-*Sc*ABF2	*Sc*Abf2p (Δ1–26) lacks import presequence, 6×His-GST tag	*E. coli* Rosetta 2 (DE3)	this study
pOPIN-3C-HALO7-PEO1	Twinkle helicase (Δ1–42) lacks import presequence, 6×His-Halo7 tag	*E. coli* Rosetta 2 (DE3)	this study
pOPINTRX-MRPL32	*h*MrpL32 (Δ1–39) lacks import presequence, 6×His-TRX tag	*E. coli* Rosetta 2 (DE3)	this study

Table S3: Yeast strains used in this study

Yeast strain	Protein version	Host	Source
JK93d	*Sc*Lon	*S. cerevisiae*	J.M. van Dijl[1]
pSEYc68-*Sc*LON	*Sc*Lon 6×His tag	*S. cerevisiae* ΔLON	J.M. van Dijl[1]

Table S4: DNA substrates used for EMSA experiments

This list shows the oligonucleotides used to construct the DNA substrates for the DNA-binding assays described in this study. The schematic figure of each substrate is depicted below; the numbers indicating a given nucleotide are positioned at its 5′ end. The asterisk (*) indicates the position of the Cy3 fluorescent dye. All substrates were prepared as described in Matulova *et al.*[3].

**Primer**	**Sequence 5′ → 3′**
Oligo 1	AGCTACCATGCCTGCACGAATTAAGCAATTCGTAATCATGGTCATAGCT
Oligo 2	AGCTATGACCATGATTACGAATTGCTTAATTCGTGCAGGCATGGTAGCT
Oligo 3	CTACAGTTCGTCAGGATTCC
Oligo 4	AATTCGTGCAGGCATGGTAGCT
Oligo 5	AGCTATGACCATGATTACGAATTGCTT
Oligo 6	AGCTATGACCATGATTACGAATTGCTTGGAATCCTGACGAACTGTAG
Oligo 7	GATGTCAAGCAGTCCTAAGGAATTCGTGCAGGCATGGTAGCT

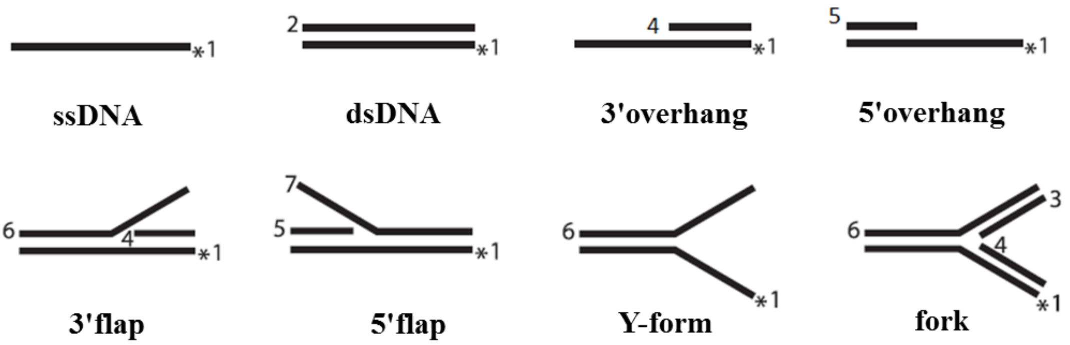

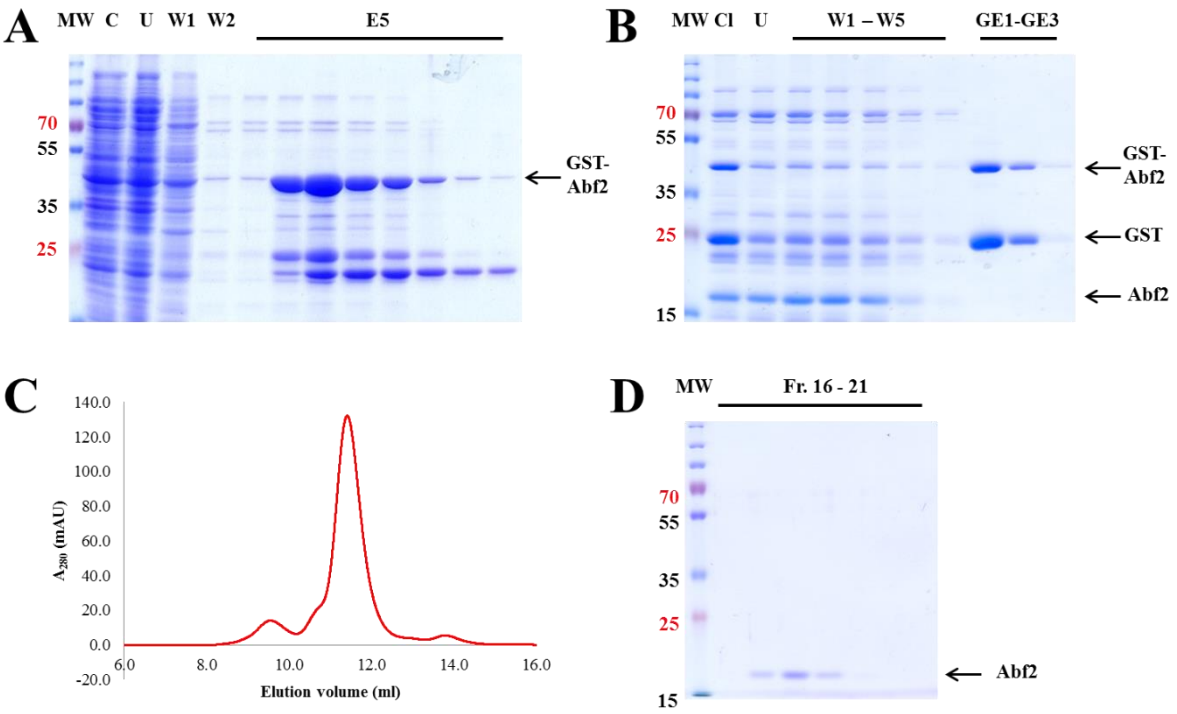

Figure S1. Isolation and purification of *Saccharomyces cerevisiae* Abf2. (A) Affinity chromatography of the Abf2 fusion protein on a Ni Sepharose 6 Fast Flow column. Elutions were made in 0.5 M imidazole (E5). (B) The His-GST tag was cleaved with PreScission protease and removed using a Glutathione Sepharose 4 Fast Flow column. (C) The sample was purified further by gel filtration on a Superdex 75 10/300 GL column. (D) The protein that eluted in 10.5–12 ml (fractions 17–19) was pooled and its concentration was determined using the BCA method. The samples from each purification step were separated in 12% SDS-PAGE gels. MW – molecular weight marker; C – cytosolic fraction; U – unbound fraction; W – wash fractions; E – elutions; Cl – cleaved sample; GE – glutathione elutions; Fr. 16–21 – SEC fractions after the separation.

A

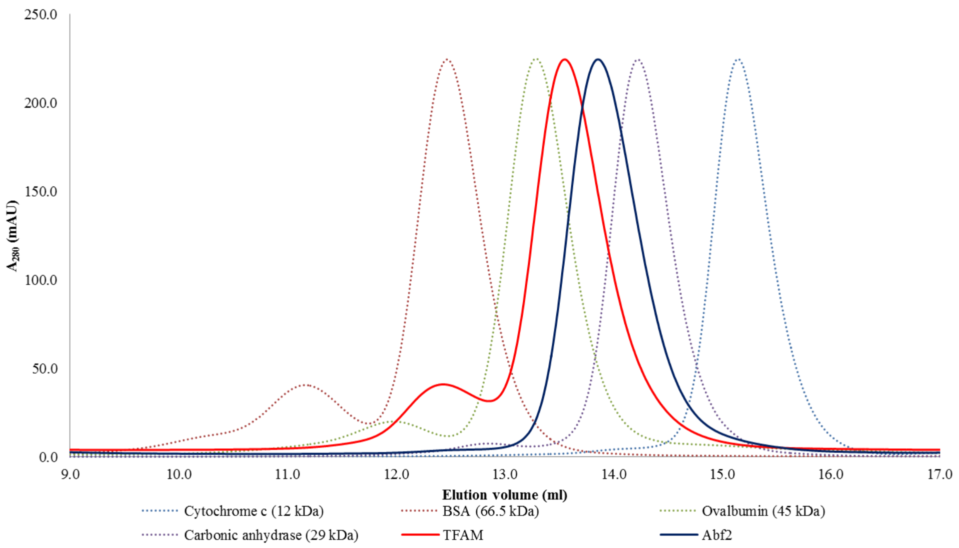

B

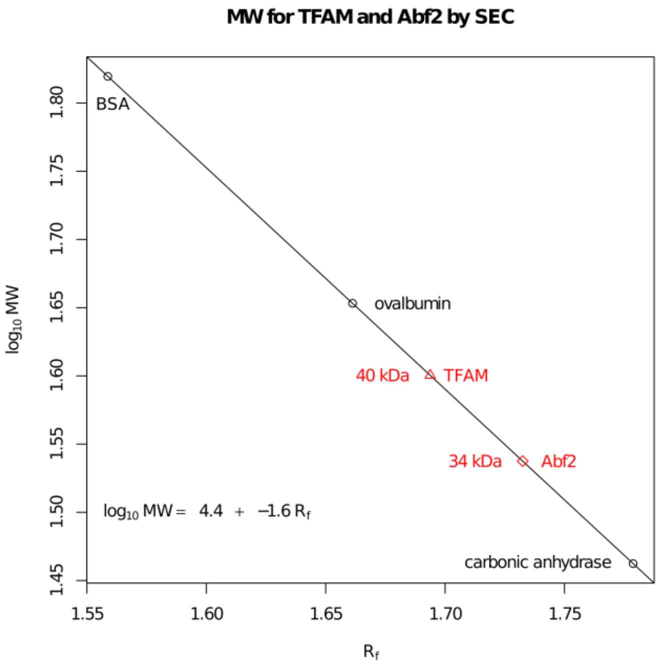

Figure S2. Determination of Abf2 and TFAM molecular weight by SEC. (A) Chromatographic comparisons of TFAM (red) and Abf2 (blue). The samples were separated on a Superose 12 10/300 GL column (GE Healthcare) equilibrated in 25 mM HEPES pH 8.0, 1 M NaCl, 5% (v/v) glycerol. The SEC elution profiles are normalized. Cytochrome *c* (12 kDa), carbonic anhydrase (29 kDa), ovalbumin (45 kDa) and BSA (66.5 kDa) were used as molecular mass standards. (B) Molecular weight determination for Abf2 and TFAM by SEC. A standard curve of the logarithm of MW versus R_f was generated using the migration distance values of three standards: BSA, ovalbumin and carbonic anhydrase. According to a linear regression, the molecular weights of Abf2 and TFAM should be 34 kDa and 40 kDa, making both proteins dimers in solution.

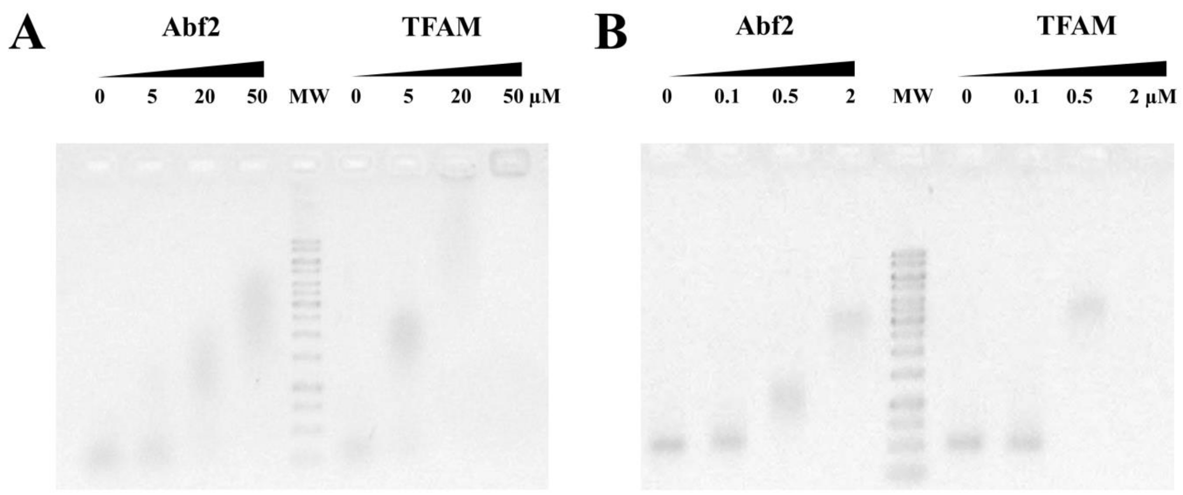

Figure S3. Comparative DNA-binding EMSA analysis of Abf2 and TFAM on (A) single- and (B) double-stranded DNA substrate. Increasing concentrations of purified protein were incubated with 10 μM 72-bp ssDNA and 4 μM 750-bp dsDNA, the same DNA probes used in the Lon-mediated digestion experiments described later. Samples were separated in a 1% agarose gel in 0.5× TBE and visualized by UV fluorescence.

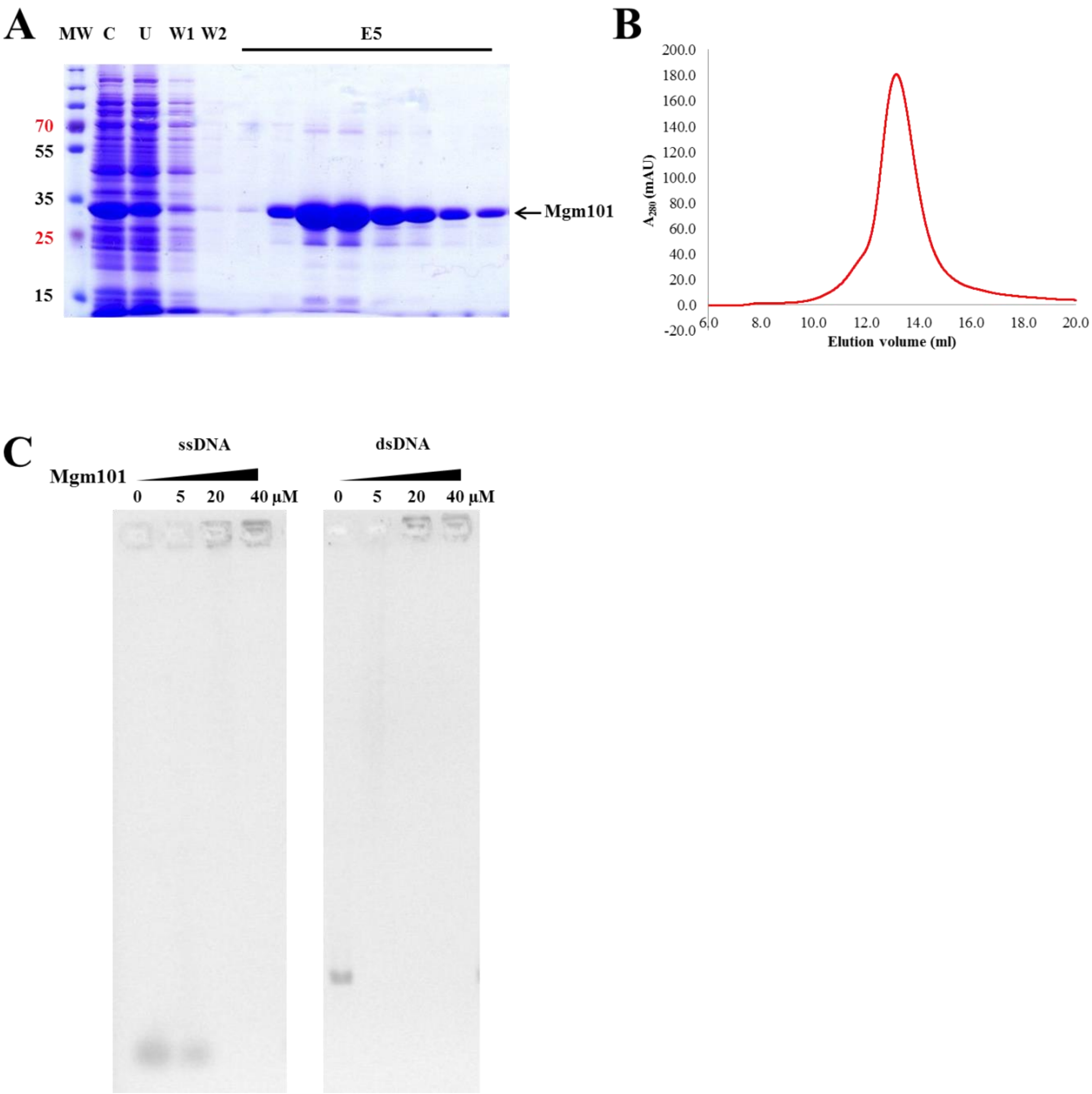

Figure S4. Isolation and functional analysis of *Saccharomyces cerevisiae* Mgm101. (A) Affinity chromatography of a 6×His-tagged recombinant Mgm101 fusion protein on a Ni Sepharose 6 Fast Flow column (GE Healthcare). Elutions were made in 0.5 M imidazole as described in Pevala *et al.*[2]. MW – molecular weight marker; C – cytosolic fraction; U – unbound fraction; W1, W2 – wash fractions; E5 – elutions. PAGE analysis was performed in a 12% SDS-polyacrylamide gel. (B) During gel filtration on a Superose 6 10/300 GL column (GE Healthcare), the protein eluted in 12.5–14.5 ml, was pooled, and its concentration was determined using the BCA method. (C) Determination of the Mgm101 DNA-binding activity on single- and double-stranded DNA substrates. Increasing concentrations of purified protein were incubated with 10 µM ssDNA and 4 µM dsDNA. Samples were separated on a 0.7% agarose gel in 0.5× TBE and visualized by UV fluorescence.

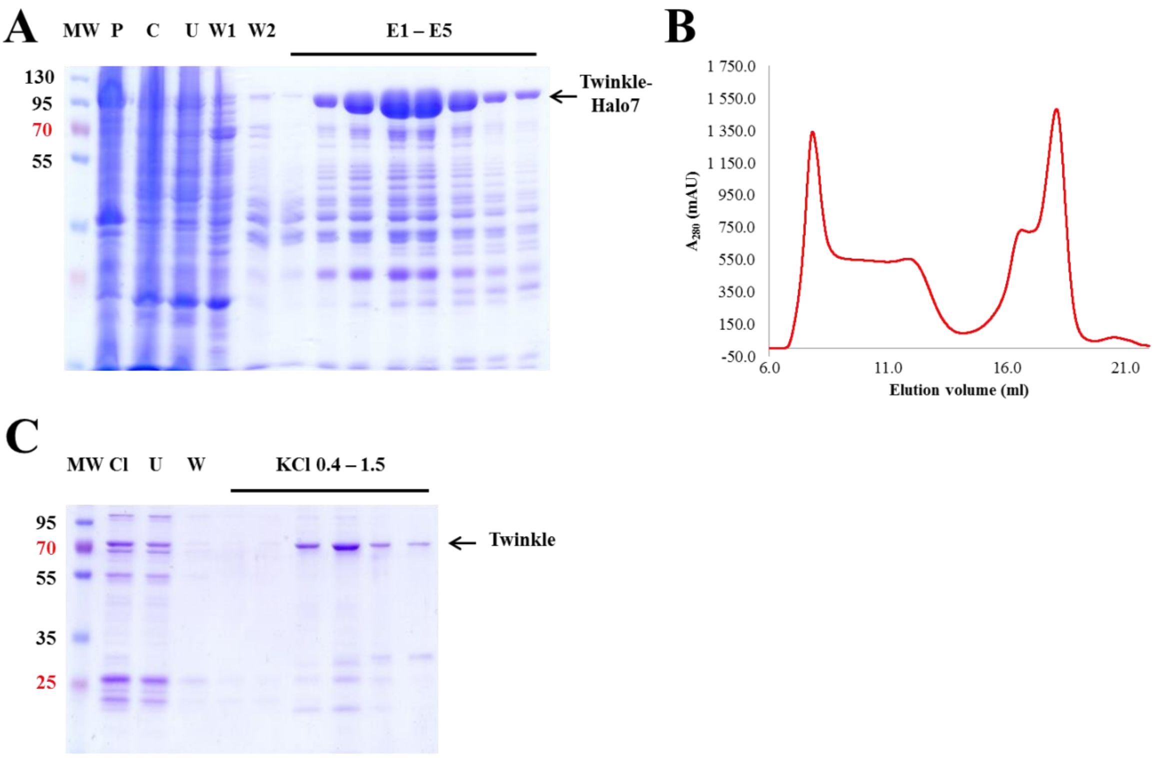

Figure S5. Isolation and purification of Twinkle helicase. (A) Affinity chromatography of the Twinkle fusion protein on a Ni Sepharose 6 Fast Flow column (GE Healthcare). Elutions were made with a stepwise imidazole gradient (0.1–0.5 M). MW – molecular weight marker; P – pellet; C – cytosolic fraction; U – unbound fraction; W1, W2 – wash fractions; E1–E5 – imidazole elutions. (B) Samples were gel filtered on a Superose 6 10/300 GL column (GE Healthcare). The protein eluted in 8.5–13.5 ml was pooled and cleaved with PreScission protease. (C) After this cleavage, the Twinkle helicase was purified on a Heparin Sepharose 6 Fast Flow column (GE Healthcare) with a stepwise KCl gradient (0.4–1.5 M). MW – molecular weight marker; Cl – cleaved sample; U – unbound fraction; W – wash fraction; KCl 0.4-1.5 – KCl elutions. Both PAGE analyses were done in 12% SDS-polyacrylamide gels.

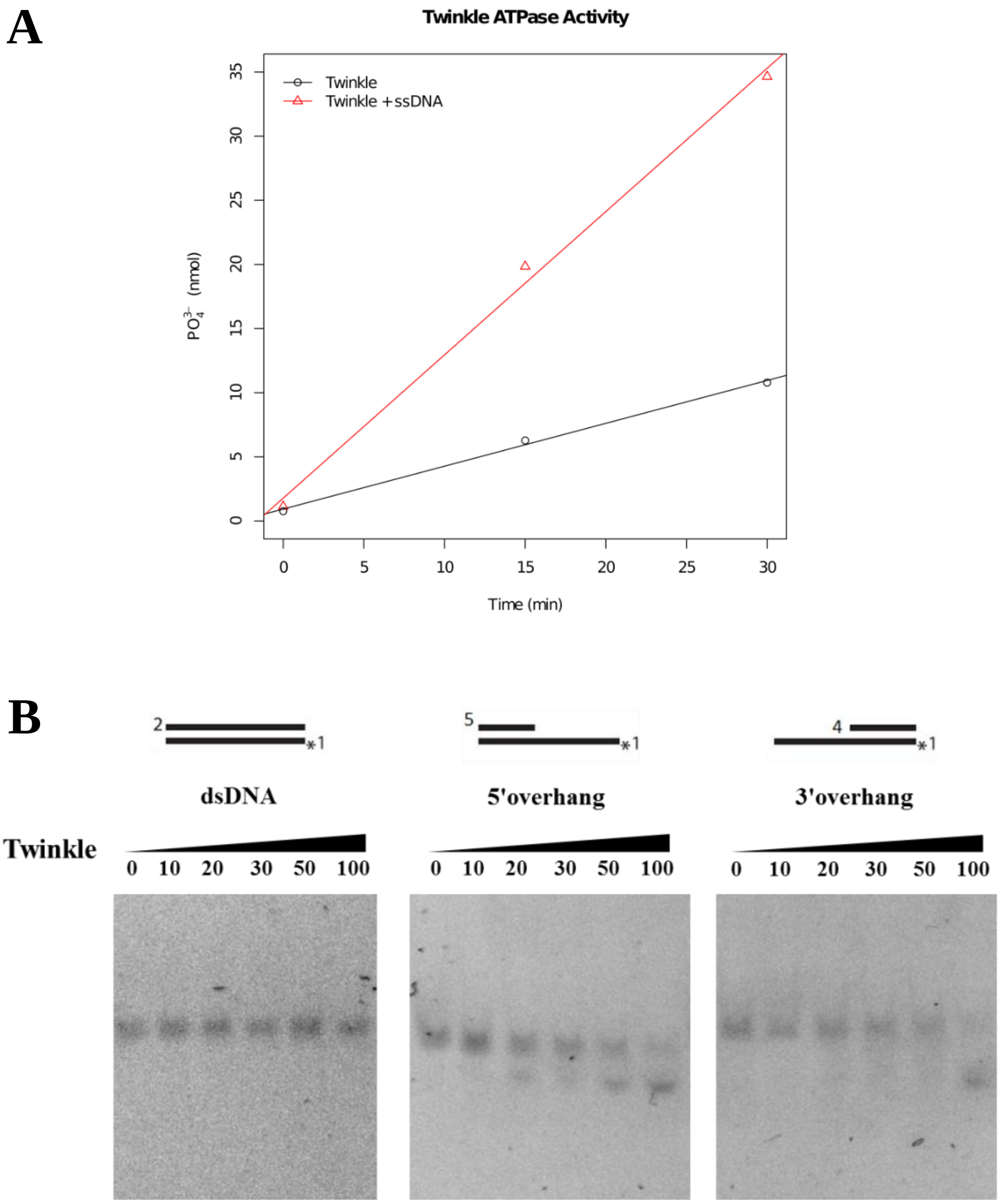

Figure S6. Twinkle helicase functional assays. (A) Quantification of Twinkle ATPase activity showing the stimulating effect of 4 μg of a single-stranded DNA probe. Each point represents an average of four independent measurements. (B) Helicase assay for the Twinkle helicase. Increasing concentrations of Twinkle (0, 10, 20, 30, 50 and 100 nM) were incubated with 75 nM fluorescently labelled DNA probes, whose structures are shown schematically above the corresponding lines. The asterisk (*) denotes the position of the Cy3 fluorescent dye. The samples were separated in 8% native polyacrylamide gels in 0.5× TBE.

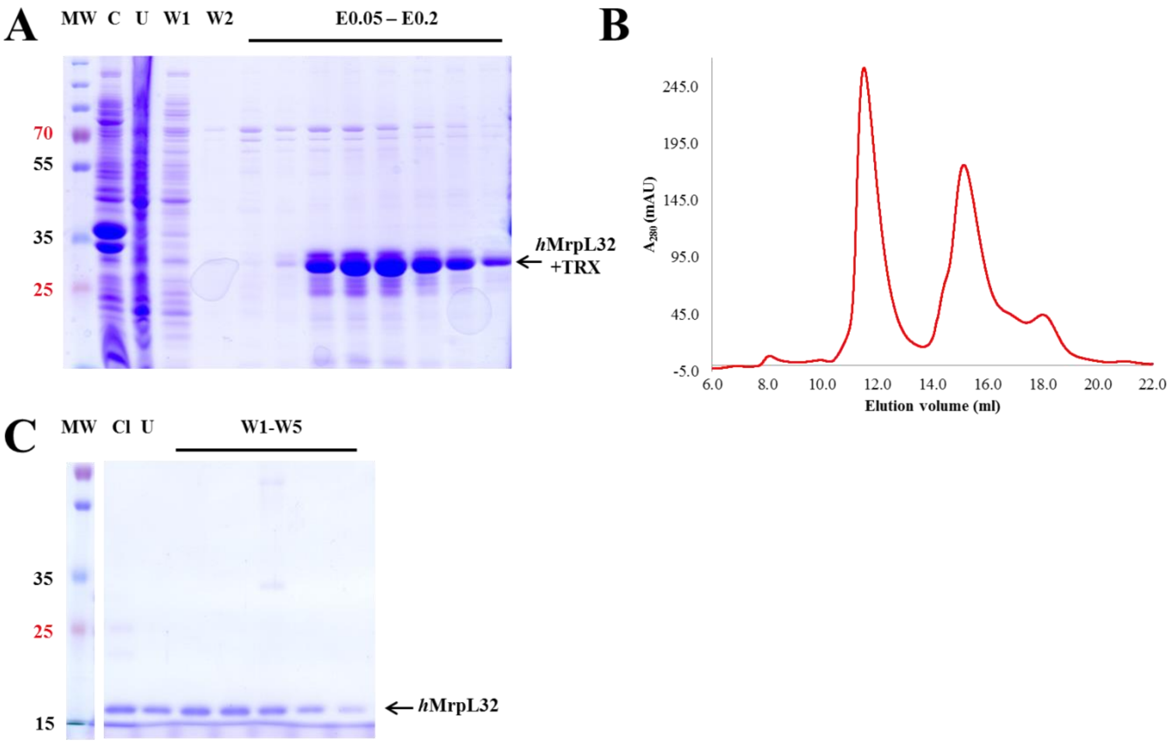

Figure S7. Isolation and purification of MrpL32. (A) Affinity chromatography of the MrpL32 fusion protein on cOmplete His-tag purification resin. Elutions were made with a stepwise imidazole gradient (0.05, 0.1, 0.15 and 0.2 M). C – cytosolic fraction; U – unbound fraction; W – wash fraction; E0.05–E0.2 – elutions; MW – molecular weight marker. (B) Elution fractions were then gel filtered on a Superdex 200 Increase 10/300 GL column (GE Healthcare). The protein eluted in 14.5–16 ml was pooled and cleaved with PreScission protease. (C) MrpL32 without the His-TRX tag was then again passed through the cOmplete His-tag resin. Cl – cleaved sample; U – unbound fraction; W1–W5 – wash fractions; MW – molecular weight marker. Both PAGE analyses of recombinant MrpL32 were done in 12% SDS-polyacrylamide gels.

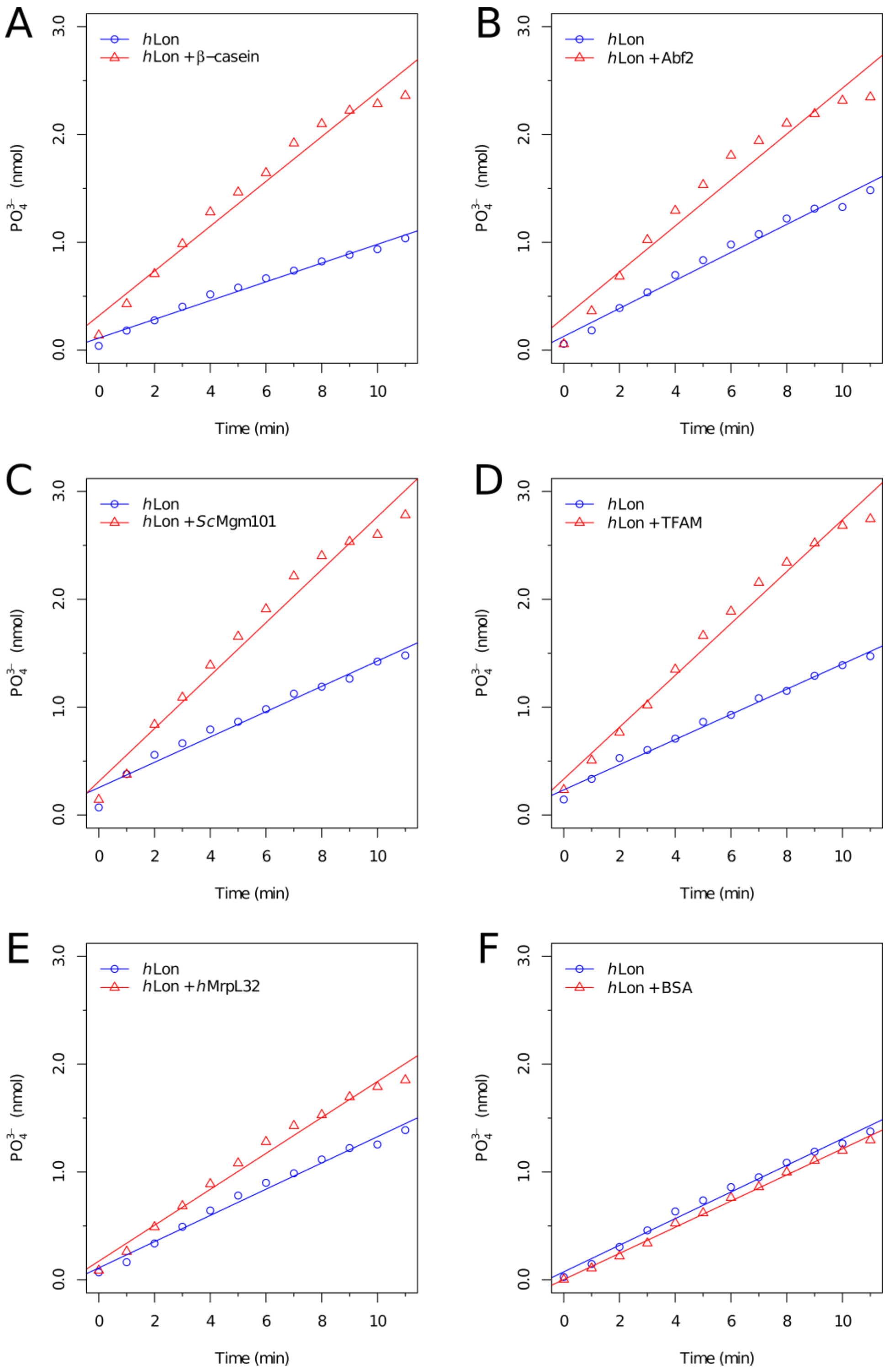

Figure S8. *h*Lon ATPase activity in the presence of protein substrates. Purified *h*Lon (5 μg) was incubated either alone or in the presence of 25 μg protein substrate (one of β-casein, Abf2, Mgm101, TFAM or *h*MrpL32) or BSA (negative control) at 37°C in reaction mixtures containing 0.5 mM ATP, 40 mM $MgCl_2$ and a colorimetric agent. The absorbance of the coloured complex formed was measured at 660 nm every 60 seconds in a 12-minute span. The values are the average of at least three independent measurements.

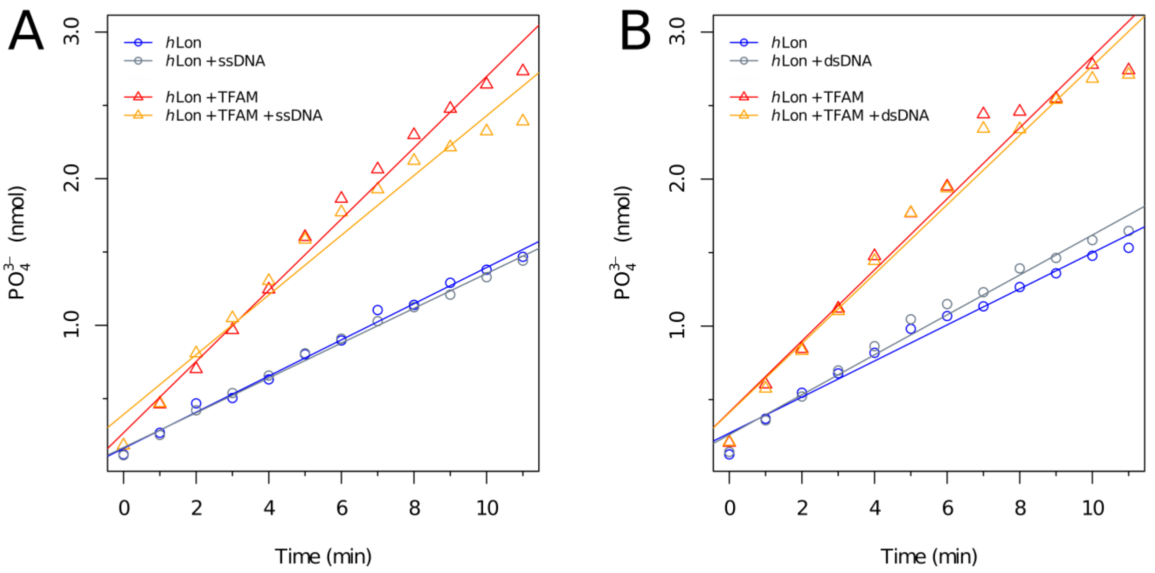

Figure S9. ATPase activity of *h*Lon in the presence of TFAM with (A) single- or (B) double-stranded DNA. Purified *h*Lon (5 µg) was incubated with 25 µg TFAM in the presence of 16 µg single-stranded or double-stranded DNA at 37°C in a reaction mixture containing 0.5 mM ATP, 40 mM $MgCl_2$ and a colorimetric agent. The absorbance of the coloured complex formed was measured at 660 nm every 60 seconds in a 12-minute span. The plots show the average of three independent measurements.

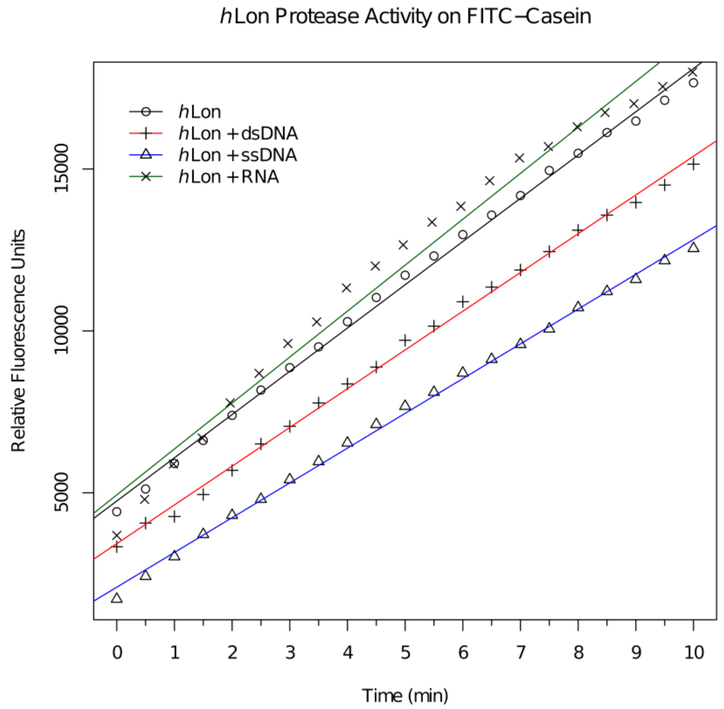

Figure S10. Protease activity of *h*Lon against FITC-casein in the presence of ssDNA, dsDNA and RNA. Purified *h*Lon (5 µg) was incubated with 15 µg FITC-casein in the presence of 16 µg single-stranded DNA, double-stranded DNA or RNA at 37°C in a reaction mixture containing 0.5 mM ATP and 40 mM $MgCl_2$. The fluorescence triggered by FITC-casein digestion was measured (excitation wavelength 492 nm, emission wavelength 530 nm) every 30 seconds in a 10-minute span. The values show the average of three independent measurements.

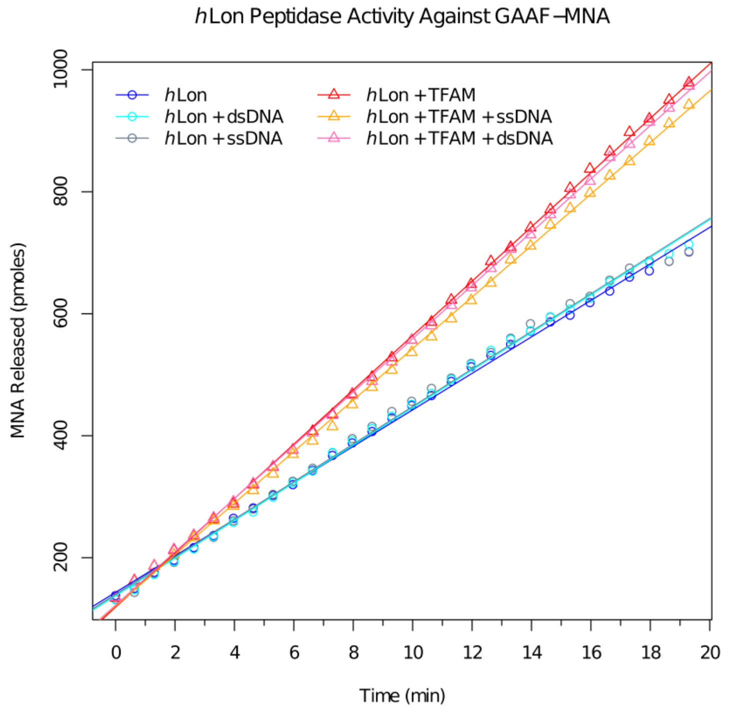

Figure S11. Peptidase activity of *h*Lon with GAAF-MNA in the presence of TFAM. Purified *h*Lon (5 µg) was incubated with 25 µg TFAM in the presence of 16 µg single-stranded or double-stranded DNA at 37°C in a reaction mixture containing 0.5 mM ATP, 40 mM $MgCl_2$ and 0.25 mM GAAF-MNA. The fluorescence triggered by GAAF-MNA cleavage was measured (excitation wavelength 340 nm, emission wavelength 400 nm) every 40 seconds in a 20-minute span. The values show the average of three independent measurements. GAAF-MNA – glutaryl-Ala-Ala-Phe-4-methoxy-β-naphthyl-amide.

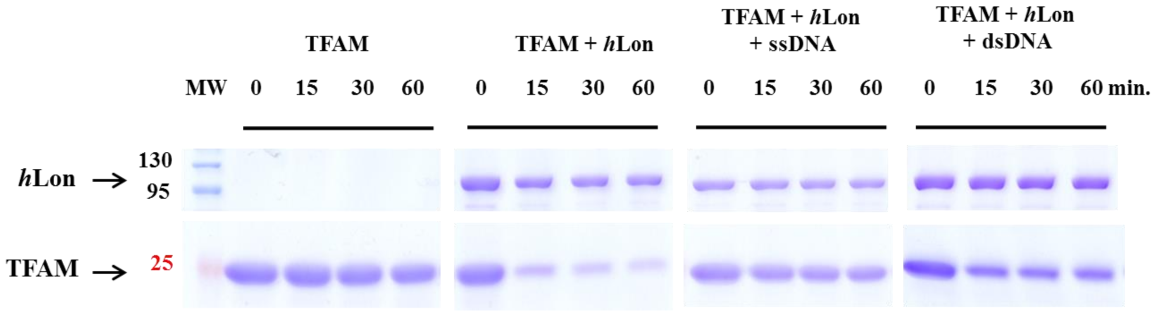

Figure S12. TFAM digestion by *h*Lon. 1.5 µg TFAM was incubated with 1 µg *h*Lon in the presence of single- and double-stranded DNA in reactions containing 2 mM ATP and 10 mM $MgCl_2$. The samples were withdrawn at the indicated times and loaded on a 12% SDS-polyacrylamide gel. MW – molecular weight marker.

A

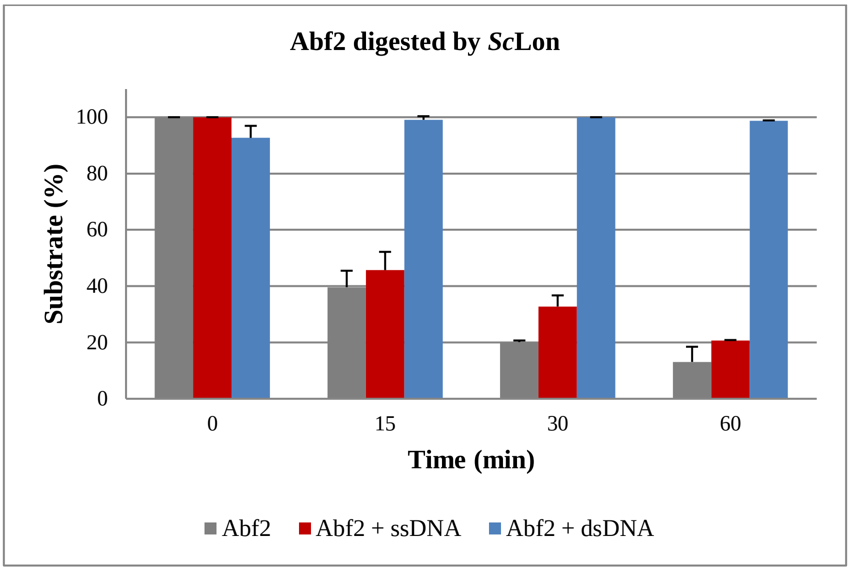
Abf2 digested by *Sc*Lon
Substrate (%)
100
80
60
40
20
0
0
15
30
60
Time (min)
Abf2
Abf2 + ssDNA
Abf2 + dsDNA

B

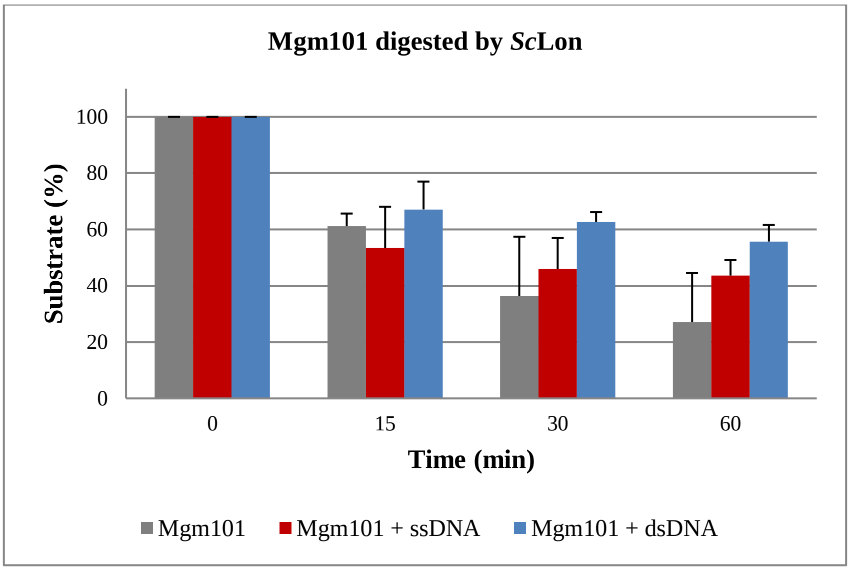
Mgm101 digested by *Sc*Lon
Substrate (%)
100
80
60
40
20
0
0
15
30
60
Time (min)
Mgm101
Mgm101 + ssDNA
Mgm101 + dsDNA

C

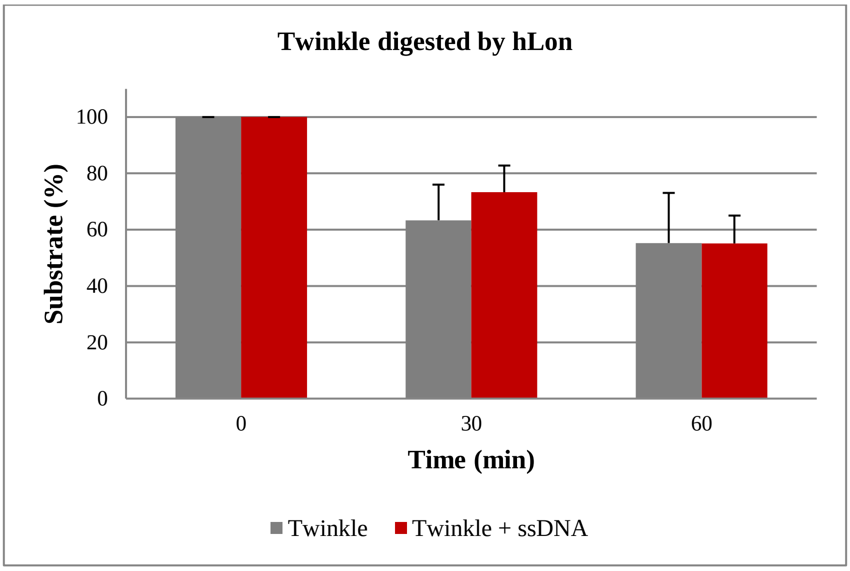
Twinkle digested by hLon
Substrate (%)
100
80
60
40
20
0
0
30
60
Time (min)
Twinkle
Twinkle + ssDNA

D

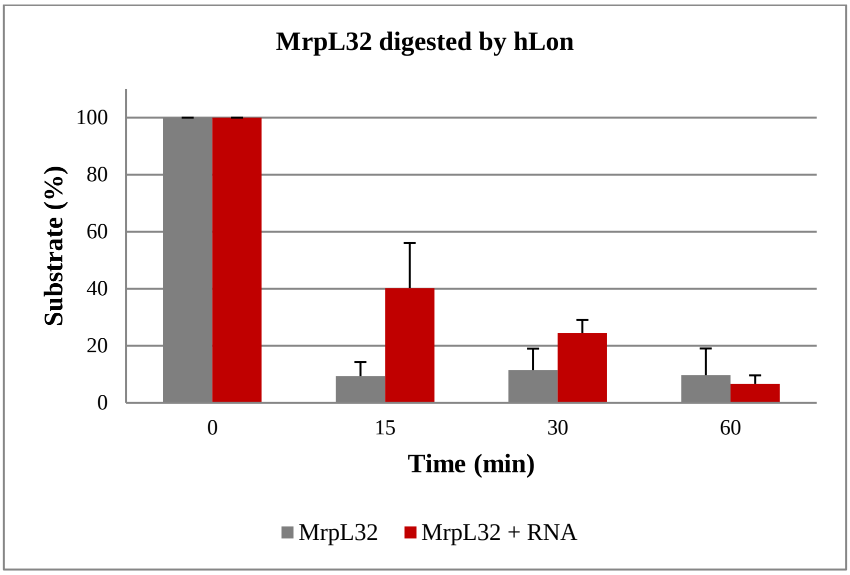

Figure S13. Quantification analysis of Lon-digestion experiments. Band intensity evaluation was performed with ImageJ separately for each studied protein. Each panel A-D arises from the gels shown, respectively, in Figures 4, 5, 7, and 8. The height of a given bar represents the mean value ± one standard deviation from at least two separate measurements.

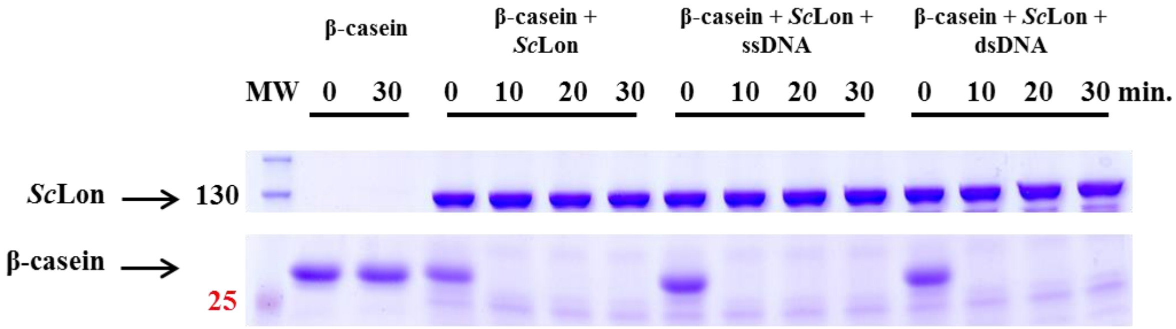

Figure S14. β-casein digestion by *S. cerevisiae* Lon. 1.5 µg β-casein was incubated with 7 µg *Sc*Lon in the presence of the given DNA probe in reaction mixtures containing 2 mM ATP and 10 mM $MgCl_2$. The samples were withdrawn at the stated times and loaded on a 12% SDS-polyacrylamide gel. MW – molecular weight marker.

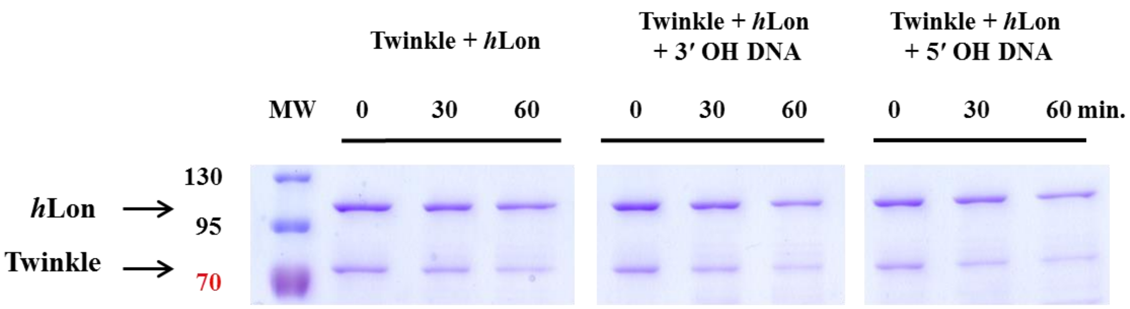

Figure S15. The course of Twinkle digestion by *h*Lon in the presence of 3′ and 5′ overhang DNA probes. 0.5 µg Twinkle was incubated with 1 µg *h*Lon either alone or in the presence of the given DNA probe in reactions containing 2 mM ATP and 10 mM $MgCl_2$. The samples were withdrawn at the indicated times and loaded on a 12% SDS-polyacrylamide gel. OH – overhang; MW – molecular weight marker.

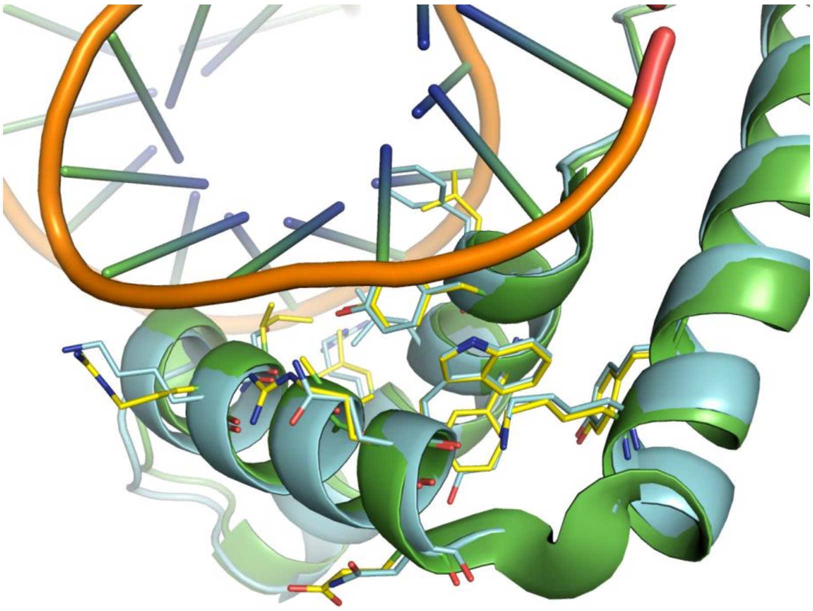

Figure S16. The HMG box of TFAM and Abf2. A superposition of the TFAM–DNA structure[4] and a homology model of Abf2 based on it showing those hydrophobic residues of the HMG box most likely to be targeted by Lon in the DNA-free form. TFAM is green with the side-chains of important residues shown as yellow sticks while the Abf2 homology model is light blue. The homology model was prepared using the Phyre2 server[5] and the picture was created using PyMOL 1.8.2[6].

BIBLIOGRAPHY

1. van Dijl, J. M. *et al.* The ATPase and protease domains of yeast mitochondrial Lon: roles in proteolysis and respiration-dependent growth. *Proceedings of the National Academy of Sciences of the United States of America* **95**, 10584-10589, (1998).
2. Pevala, V. *et al.* The structure and DNA-binding properties of Mgm101 from a yeast with a linear mitochondrial genome. *Nucleic acids research* **44**, 2227-2239, (2016).
3. Matulova, P. *et al.* Cooperativity of Mus81.Mms4 with Rad54 in the resolution of recombination and replication intermediates. *The Journal of biological chemistry* **284**, 7733-7745, (2009).
4. Rubio-Cosials, A. *et al.* Human mitochondrial transcription factor A induces a U-turn structure in the light strand promoter. *Nature structural & molecular biology* **18**, 1281-1289, (2011).
5. Kelley, L. A., Mezulis, S., Yates, C. M., Wass, M. N. & Sternberg, M. J. The Phyre2 web portal for protein modeling, prediction and analysis. *Nature protocols* **10**, 845-858, (2015).
6. Schrodinger, L. The PyMOL Molecular Graphics System, Version 1.8. (2015).
